# Intrinsically disordered linkers determine the interplay between phase separation and gelation in multivalent proteins

**DOI:** 10.7554/eLife.30294

**Published:** 2017-11-01

**Authors:** Tyler S Harmon, Alex S Holehouse, Michael K Rosen, Rohit V Pappu

**Affiliations:** 1 Center for Biological Systems Engineering, Department of Biomedical EngineeringWashington University in St. LouisSt. LouisUnited States; 2 Department of BiophysicsHoward Hughes Medical Institute, UT Southwestern Medical CenterDallasUnited States; Max Planck Institute of Molecular Cell Biology and GeneticsGermany

**Keywords:** phase transitions, phase separation, gelation, intrinsically disordered proteins, multivalent proteins, computation, None

## Abstract

Phase transitions of linear multivalent proteins control the reversible formation of many intracellular membraneless bodies. Specific non-covalent crosslinks involving domains/motifs lead to system-spanning networks referred to as gels. Gelation transitions can occur with or without phase separation. In gelation driven by phase separation multivalent proteins and their ligands condense into dense droplets, and gels form within droplets. System spanning networks can also form without a condensation or demixing of proteins into droplets. Gelation driven by phase separation requires lower protein concentrations, and seems to be the biologically preferred mechanism for forming membraneless bodies. Here, we use coarse-grained computer simulations and the theory of associative polymers to uncover the physical properties of intrinsically disordered linkers that determine the extent to which gelation of linear multivalent proteins is driven by phase separation. Our findings are relevant for understanding how sequence-encoded information in disordered linkers influences phase transitions of multivalent proteins.

## eLife digest

Our cells contain a variety of structures called organelles that perform specific roles within a cell. Some organelles are surrounded by a membrane, while others float inside the cell as spherical droplets made of proteins. These proteins contain several sticky regions, which are connected by flexible linker proteins. It is thought that the level of stickiness and the number of sticky regions, or domains, determine whether a protein will form a membraneless organelle. Often, proteins with similar sticky domains have different linkers, and until now, it was assumed that the linkers do not have any other purpose than stringing the domains together.

To test this further, Harmon et al. used a combination of computer simulations and physics-based theory. In these simulations, the domains were kept the same, but the properties of linkers were changed to see if this would influence how the membraneless organelles are formed.

The results showed that depending on the physical properties of the linkers, the proteins could huddle together and form dense spherical gel-like droplets similar to the membraneless organelles, or form open non-spherical gels. When the linkers were short, the proteins do not easily form droplets. Linkers that were sufficiently long but too bulky, lead to non-spherical gels. Compact linkers, however, enabled proteins to huddle and form spherical gels. The spherical droplet-spanning gels required much less protein compared to the open non-spherical gels.

This suggests that proteins important for forming membraneless organelles can be distinguished from those that are not based on the properties of their linkers – even when their domains are similar.

These findings further scientists’ knowledge of how specific types of proteins form membraneless organelles and will help to understand how membraneless organelles control many key aspects of how a cell works.

## Introduction

**Figure 1.**
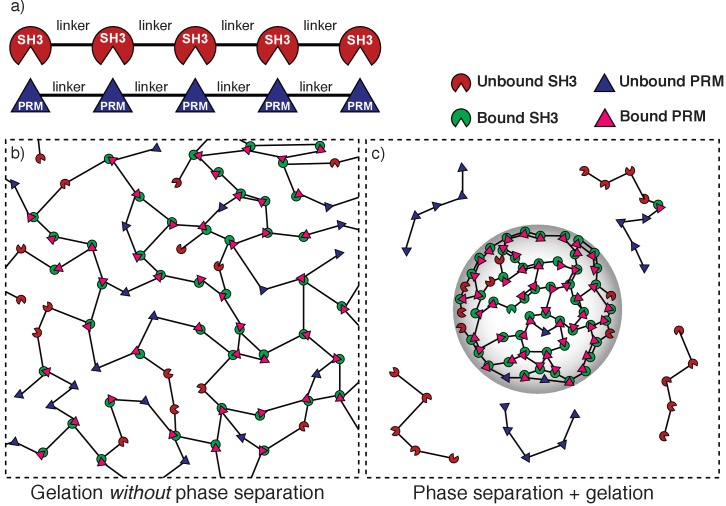
Depiction of gelation without phase separation as opposed to phase separation plus gelation. (**a**) Schematic of a synthetic multivalent system. SH3 domains bind to proline-rich modules (PRMs). Multivalent SH3 and PR proteins result from the tethering of multiple SH3 domains (or PRMs) by linkers. (**b**) Schematic of gelation without phase separation: If the bulk concentration of interaction domains is above the gel point but below the saturation concentration then a system spanning network forms across the entire system volume. In this scenario, a percolation transition is realized without phase separation. (**c**) Schematic of phase separation plus gelation. Linker-mediated cooperative interactions of multivalent proteins drive phase separation, depicted here as a confinement of molecules into a smaller volume (gray envelope) when compared to the system volume (dashed bounding box). If the bulk concentration of interaction domains is higher than a saturation concentration then a dense phase comprising of multivalent SH3 and PRM proteins will be in equilibrium with a dispersed phase of unbound proteins. A droplet-spanning network will form because the concentration of interaction domains within the dense phase is above the gel point.

There is growing interest in intracellular phase transitions that lead to the formation of membraneless bodies that are collectively known as biomolecular condensates (Banani et al., 2017; Shin and Brangwynne, 2017). These are two- or three-dimensional assemblies that comprise of multiple proteins and RNA molecules and lack a surrounding membrane. Biomolecular condensates are associated with a range of cellular functions including cell signaling (Su et al., 2016), ribosomal biogenesis (Feric et al., 2016; Zhu and Brangwynne, 2015; Mitrea et al., 2016), cytoskeletal regulation (Li et al., 2012; Banjade and Rosen, 2014), stress response (Parry et al., 2014; Munder et al., 2016; Ramaswami et al., 2013; Riback et al., 2017), cell polarization (Saha et al., 2016; Nott et al., 2015), and cytoplasmic branching (Lee et al., 2015). It has been proposed that the protein components of biomolecular condensates can be classified as scaffolds versus clients (Banani et al., 2017; Banani et al., 2016). Scaffolds are thought to drive phase transitions, whereas client molecules preferentially partition from the cytoplasm or nucleoplasm into condensates (Banani et al., 2016; Wheeler et al., 2016). Scaffold proteins that drive phase transitions have distinct features, the most prominent being *multivalency* of folded domains or **S**hort **Li**near amino acid **M**otifs (SLiMs) (Banani et al., 2017; Li et al., 2012; Brangwynne et al., 2015; Csizmok et al., 2016; Kato et al., 2012). Valency quantifies the number of interaction domains or SLiMs. Ligands of multivalent proteins can be other multivalent proteins or polynucleotides. The simplest multivalent proteins are linear polymers that consist of multiple protein-protein/protein nucleic acid interaction domains or SLiMs connected by intrinsically disordered linkers that lack specific interaction motifs (Figure 1a).

Linear multivalent proteins may be classified as associative polymers (Tanaka, 2011; Semenov and Rubinstein, 1998), with specific intra- and intermolecular associations being mediated by non-covalent interactions amongst domains or motifs. Unlike generic homopolymers where the interactions are isotropic, uniform, and typically short-range (Flory, 1942a; Flory, 1974), the interactions involving associative polymers span a range of length scales and can be directional in nature (Brangwynne et al., 2015; Tanaka, 2011). This includes a hierarchy of so-called weakly polar interactions involving charges, dipoles, and quadrupoles (Nott et al., 2015; Brangwynne et al., 2015; Burley and Petsko, 1988; Brady et al., 2017; Lin et al., 2016), hydrogen bonds, screened charge-charge interactions (Pak et al., 2016), and hydration-mediated interactions (Boeynaems et al., 2017; Schneider et al., 2002; Pochan et al., 2003). This hierarchy of interactions will enable non-covalent interactions known as physical crosslinks that involve associative domains/motifs that enable the formation of system-spanning networks known as gels (Semenov and Rubinstein, 1998; Schneider et al., 2002; Pochan et al., 2003; Rubinstein and Colby, 2003). Associative polymers can undergo two types of reversible gelation transitions. These are *gelation without phase separation* or *gelation driven by phase separation* (Tanaka, 2011; Semenov and Rubinstein, 1998; Rubinstein and Semenov, 1998). Our work focuses on the differences between the distinct gelation transitions and the molecular determinants of these differences in linear multivalent proteins.

*Gelation without phase separation* refers to a switch from a solution of dispersed monomers and oligomers – a sol – to a system-spanning network – a gel (Figure 1b). This *networking transition* is characterized by a concentration threshold, known as the percolation threshold (Broadbent and Hammersley, 1957) that defines the *gel point* (Flory, 1941, 1942b; Stockmayer, 1943). If the bulk concentration of associative domains/motifs is below the gel point, then the multivalent proteins form a sol. For concentrations above the gel point, the multivalent proteins are incorporated into a system-spanning network known as a physical gel.

Physical gels (referred to hereafter as *gels*) are defined by specific, reversible non-covalent interactions, that represent physical crosslinks between protein modules/SLiMs and their ligands (Su et al., 2016; Tanaka, 2011; Falkenberg et al., 2013). Therefore, a gel is a percolated network characterized by system-spanning reversible physical crosslinks. Accordingly, the average extent of crosslinking will determine the network structure including the free volume or porosity, and average stiffness of the gel. Conversely, the timescales for making and breaking crosslinks will determine the rheological properties of gels (Tanaka, 2011). This definition of a gel, which is based on Flory’s work (Flory, 1974), is also consistent with criteria outlined by Almdal et al. (Almdal et al., 1993). It is important to clarify that our definition of a gel does not conflate gels with solids nor does it suggest that gels have to be pathological states of matter.

Polymer solutions can also undergo *phase separation* (Brangwynne et al., 2015; Flory, 1942a; Pak et al., 2016; Huggins, 1942). Above a saturation concentration, the polymer solution will undergo phase separation by separating into a dense polymer-rich phase that coexists with a dilute liquid that is deficient in polymers (Flory, 1942a; Huggins, 1942). The formation of two coexisting phases characterized by phase separation represents a condensation or *density transition*, with the dense phases forming spherical droplets (Figure 1c). Given the three-way interplay among polymer-solvent, solvent-solvent, and polymer-polymer interactions, a necessary condition for phase separation is that inter-polymer attractions are more favorable, on average, when compared all other interactions (Brangwynne et al., 2015; Tanaka, 2011; Rubinstein and Colby, 2003).

Interestingly, phase separation of associative polymers such as linear multivalent proteins will promote gelation if the concentration of interaction domains within the dense phase is above the gel point (Figure 1c). To understand this conceptually, we shall denote *c_g_* as the gel point whilst *c_sl_* and *c_sh_* will respectively denote the saturation concentrations of the coexisting dilute and dense phases that result from phase separation. For *gelation without phase separation* the gel point lies below the saturation concentration for phase separation (*c_g_* >*c_sl_*). In contrast, if *c_sl_* <*c_g_* < *_csh_* the gel point lies below the saturation concentration for phase separation and the concentration within the coexisting dense droplet is above the gel point. In this scenario, the system will undergo *gelation driven by phase separation* thus resulting in droplet-spanning networks.

*What are the molecular determinants of gelation with and without phase separation*? We answer this question by focusing on linear multivalent proteins with folded domains interspersed by disordered linkers. Specifically, using computer simulations and theoretical analysis we show that for linear multivalent proteins of fixed binding affinity between modules and valence, the disordered linkers determine the preference for gelation driven by phase separation as opposed to gelation without phase separation. This behavior is determined by the sequence-specific properties of linkers, which can be quantified in terms of a single parameter known as the effective solvation volume (v_es_).

**Figure 2.**
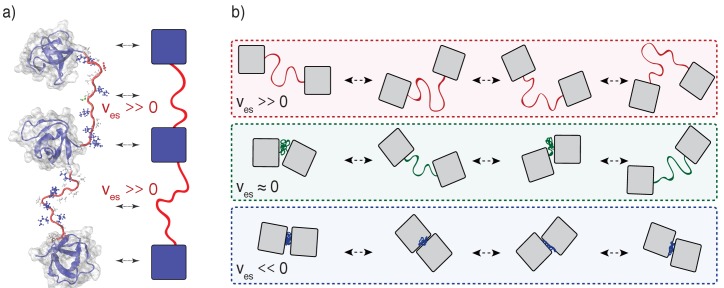
Illustration of the impact of linker effective solvation volumes on the conformational fluctuations and inter-domain distances in linear multivalent proteins. (**a**) Schematic of three SH3 domains connected by positive v_es_ linkers. In a cartoon schematic, the SH3 domains are shown as blue squares and the linkers are depicted as red tethers. The bidirectional arrows indicate the mapping between the molecular structures and the cartoon schematic. (**b**) Comparative schematics of SH3 domains connected by different types of linkers. The top row shows a pair of domains connected by linkers of high positive effective solvation volumes. For linkers with near zero effective solvation volumes, the inter-domain distances are characterized by large fluctuations and this engenders large concentration fluctuations. The bottom row shows the scenario for domains connected by linkers with negative v_es_ values. In this scenario, the inter-domain distances seldom exceed the sum of the individual radii of gyration.

The effective solvation volumes reflect the average volumes occupied by linkers, referenced to the volume occupied if that linker lacked a bias to be well solvated or poorly solvated (Rubinstein and Colby, 2003). When residues prefer to interact with solvent several additional layers of solvent effectively bloat them, and so the linker becomes expanded. When residues prefer to interact with other residues they have less volume for the solvent, and so the linker becomes compact. The effective solvation volume (v_es_) of a linker can be pictured in terms of the impact that a linker has on bringing together interaction modules that are connected to either end (see Figure 2). Qualitatively, we can think about this in terms of a hypothetical outwards force that acts on the two interaction modules at either end of the linker. When v_es_ is positive, the linker is highly expanded and this outwards force repels the two interaction modules, driving them apart. A positive v_es_ is realized because the linker is self-repelling, carving for itself a large volume in space for favorable interactions with the solvent. When v_es_ is negative, the linker is compact, and the hypothetical force pulls the two interaction modules inward, driving them close together. A negative v_es_ is realized because the solvent is squeezed out, the linker is self-attractive, and this causes the interaction domains to be pulled towards one-another. When v_es_ is close to zero, the linker does not have strong interaction preferences and mimics a passive tether. Accordingly, both expanded and compact linker conformations are equally likely. The hypothetical outwards/inward force is negligible – the preferences for compact versus expanded conformations cancel one another – and the interaction modules meander around in three-dimensional space with respect to one another, restrained only the connectivity of the linker. A value of v_es_ ≈ 0 is realized due to a counterbalancing of attractive and repulsive interactions in the linker.

Formally, the effective solvation volume of a linker is quantified in terms of the solvent-mediated pairwise interactions between pairs of linker residues and the details are discussed in Appendix 1. If the linker sequence is such that there are *net* attractions between all pairs of residues, then v_es_ will be negative and this will be true for linkers that form compact globules. Conversely, if there are *net* repulsions between all pairs of residues, then the residues prefer to be solvated and v_es_ will be positive. This is the case for so-called self-avoiding random coil (SARC) linkers. Finally, if the effects of inter-residue attractions offset the effects of inter-residue repulsions, then v_es_ ≈ 0 and this is the scenario for so-called Flory random coil (FRC) linkers. The effective solvation volume is directly proportional to the second virial coefficient denoted as *B*_2_ (32, 41). Negative, zero, or positive values of v_es_ correspondingly imply negative (attractive interactions), zero (non-interacting), or positive (repulsive interactions) values of *B*_2_. Therefore, v_es_ can be inferred using either atomistic simulations (as shown in this work) or via measurements of *B*_2_ as shown by Wei et al. (Wei et al., 2017).

For generic homopolymers, the sign and magnitude of v_es_ are determined by the effective chain-solvent interactions, which in turn depend on the chemical makeup of the chain. For proteins, the interplay between chain-chain and chain-solvent interactions is specified by the amino acid sequence, whereby the composition and patterning of a disordered linker will determine the balance of chain-chain and chain-solvent interactions (Das et al., 2015; Holehouse et al., 2017; Martin et al., 2016). Therefore, the effective solvation volume of a disordered linker is determined directly by its primary sequence.

To set the stage for our investigations, we first performed proteome-wide bioinformatics analysis combined with all-atom simulations to quantify conformational consequences of sequence-specific effective solvation volumes of disordered linkers in naturally occurring multi-domain human proteins. This analysis shows that the sub-proteome of linear multivalent proteins comprises of linkers of varying lengths that span a range of effective solvation volumes, from significantly negative to significantly positive values. Using coarse-grained numerical simulations and analytical theories we then show that the type of gelation transitions that linear multivalent proteins undergo is directly determined by the physical properties of linkers, which include the lengths of linkers and their sequence-specific effective solvation volumes.

## Results

### Disordered linkers between folded domains in the human proteome span the entire range of effective solvation volumes

**Figure 3.**
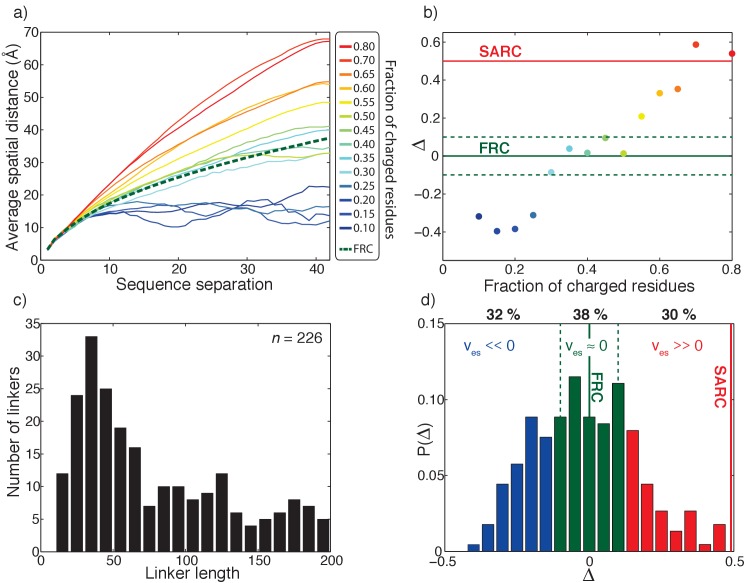
Effective solvation volumes for disordered linkers from the human proteome. (**a**) Inter-residue distance profiles for fourteen representative sequences, each 40-residues long. The legend shows the fraction of charged residues within each linker. The green dashed curve shows the inter-residue distance profile for the reference FRC limit. (**b**) Summary of the variation of ∆ as a function of the fraction of charged residues for the fourteen representative sequences. Here, ∆ $�=\frac{1}{N}\sum_{k}\frac{\left\langle R_{k}\right\rangle -\left\langle R_{k}^{\text{FRC}}\right\rangle }{\left\langle R_{k}^{\text{FRC}}\right\rangle }$, *N* is the number of linker residues,$\left\langle R_{k}\right\rangle$is the average spatial separation between residue pairs that are *k* apart in the linear sequence, $\left\langle R_{k}^{\text{FRC}}\right\rangle$is the corresponding spatial separation for a FRC chain, and the summation index *k* runs across all sequence-separations. Linkers for which ∆ < –0.1 will have negative effective solvation volumes (v_es_ < 0); linkers for which –0.1 ≤ ∆≤0.1 will have near zero effective solvation volumes (v_es_ ≈ 0); and linkers for which ∆>0.1, will have positive effective solvation volumes (v_es_ > 0). For the self-avoiding random coil (SARC) linkers, ∆ ≈ 0.5 and this is shown as a horizontal red line. (**c**) Length distribution of all 226 unique disordered linkers. (**d**) Distribution of ∆ values extracted from all-atom simulations of all 226 linkers. Based on the results shown in panel (**B**), we delineate the ∆-distribution into three regimes: ∆ < –0.1 (blue bars), –0.1 ≤ ∆≤0.1 (green bars), and ∆>0.1 (red bars). These regimes correspond, respectively to linkers for which v_es_ is less than zero, near zero, or greater than zero.

We first sought to obtain accurate and efficient estimates of the effective solvation volume (v_es_) for a large set of disordered segments. For this we used all-atom simulations, which have a proven track record of describing sequence-specific conformational properties of intrinsically disordered proteins (Das et al., 2015; Martin et al., 2016; Vitalis and Pappu, 2009a2009; Das et al., 2016). Although a formal and rigorous calculation of v_es_ is technically possible using these simulations, this approach is computationally expensive and non-trivial for large numbers of sequences. Recognizing that the effective solvation volume directly determines the global dimensions of a linker, we used the ensemble-averaged conformational properties to calculate a proxy for v_es_ (Mao et al., 2013). Specifically, we leverage the profile of inter-residue distances to determine how a given linker sequence deviates from a sequence-specific theoretical reference that recapitulates v_es_ = 0, which is the Flory Random Coil (FRC) (Holehouse et al., 2015). These profiles (Figure 3a) describe the average spatial separation between all pairs of residues as a function of their separation along the polypeptide sequence.

We obtained sequence-specific inter-residue distance profiles by performing all-atom Metropolis Monte Carlo simulations using the ABSINTH implicit solvent model and forcefield paradigm (Vitalis and Pappu, 2009b) as described in the methods section. Figure 3a shows the calculated inter-residue distance profiles for fourteen distinct sequences, each of length 40 residues. Details of the sequences are shown in (Table 1). Figure 3a illustrates changes to the inter-residue distance profiles as a function of changes to the fraction of charged residues. Figure 3a also shows the inter-residue distance profile for a reference FRC linker. Sequences with positive v_es_ will have inter-residue distance profiles that lie above the FRC reference. Conversely, sequences with negative v_es_ will have profiles with uniformly smaller inter-residue spatial separations for given sequence separations when compared to the FRC reference. Accordingly, Figure 3a shows that sequences deficient in charged residues are expected to have negative v_es_ values, whereas sequences enriched in charges are expected to have positive v_es_ values.

**Table 1.** Summary of the parameters, the physical description of these parameters, and the default values used for the parameters of the lattice model.

Parameter	Physical interpretation	Default value
Valence	Number of PRMs and SH3 domains per poly-PRM and poly-SH3	5 (but titrated for results in Figure 6)
Interaction Strength	Intrinsic affinity between PRMs and SH3 domains	–2*k_B_T*
Linker Length	Length of disordered linker between interaction domains	5 (but titrated for results in Figure 8)
Effective solvation volume (v_es_)	Degree to which the Linker Prefers Interacting with Solvent	Proportional to the number of explicitly modeled linker beads

**Table 2.** Details of the fourteen sequences chosen at random from the human proteome. All sequences have identical lengths (40 residues) and are enriched in disorder promoting residues. The sequences are listed in descending order of the fraction of charged residues.

Sequence	FCR*	NCPR^†^	Fraction of disorder promoting residues	UNIPROT identifier of protein from which the sequence was drawn
EDEDSEKEEEEEDKEMEELQEEKECEKPQGDEEEEEEEEE	0.80	–0.60	0.93	P37275
DEEGNAYGSEREEEDEEEDEEDGKRELELEEEELGGEEED	0.70	–0.55	0.88	P78415
REKDREKYSQREQERDRQQNDQNRPSEKGEKEEKSKAKEE	0.65	0.00	0.93	Q9H0G5
DRVVVTDDSDERRLKGAEDKSEEGEDNRSSESEEESEGEE	0.60	–0.30	0.88	Q9BQG0
EAYRLSLEADRAKREAHEREMAEQFRLEQIRKEQEEEREA	0.55	–0.10	0.88	Q9UNN5
RRQRRWEDIFNQHEEELRQVDKDKEDESSDNDEVFHSIQA	0.50	–0.15	0.73	Q7Z2Y5
NNRKGRGGNRGREFRGEENGIDCNQVDKPSDRGKRARGRG	0.45	0.15	0.76	Q5T6F2
QKQKLRLLSSVKPKTGEKSRDDALEAIKGNLDGFSRDAKM	0.40	0.10	0.75	Q9UMZ2
AEMKVLESPENKSGTFKAQEAEAGVLGNEKGKEAEGSLTE	0.35	–0.10	0.78	Q8N3D4
MAAAESDKDSGFSDGSSECLSSAEQMESEDMLSALGWSRE	0.30	–0.20	0.78	Q9C0C6
DHFMKSGFASGRNFGNRDAGECNKRDNTSTMGGFGVGKSF	0.25	0.05	0.68	Q9NQI0
TAVSTSGPEDICSSSSSHERGGEATWSGSEFEVSFLDSPG	0.20	–0.15	0.80	Q9BQQ3
FSTLGRLRNGIGGAAGIPRANASRTNFSSHTNQSGGSELR	0.15	0.10	0.73	Q9Y252
KSSSQTSGSLVSKSTSLASVSQLASKSSSQTSTSQLPSKS	0.10	0.10	0.85	Q9NXV6

*FCR: Fraction of charged residues defined as (*f*_+_+*f*_–_) where *f*_+_ and *f*_–_ denote the fraction of positive and negative charges, respectively;. †NCPR: Net charge per residue defined as (*f*_+_ – *f*_–_).

Since inter-residue distance profiles are direct manifestations of sequence-specific effective solvation volumes (Mao et al., 2013), we use these profiles to calculate a parameter ∆ that serves as a proxy for sequence-specific v_es_ values. This parameter is defined as the mean signed difference between the sequence-specific inter-residue distance profile and the corresponding profile for a FRC reference. In Figure 3b we plot the calculated ∆ values against the fraction of charged residues for the fourteen disordered sequences from Figure 3a. The value of ∆ can be negative, equal to zero, or positive and this depends on whether the value of v_es_ is negative, zero, or positive, respectively.

Sequences that form compact globules have negative values of v_es_ and negative values of ∆. For the sequences examined, this is true when the fractions of charged residues is below 0.3. Within an interval between 0.3 and 0.5 for the fraction of charged residues, sequences mimic the FRC limit, where v_es_ ≈ 0. This is manifest as –0.1 ≤ ∆≤0.1. Sequences that prefer chain-solvent interactions to intra-chain interactions will be expanded relative to the FRC limit. This leads to positive values of v_es_ and corresponds to values of ∆ that are greater than 0.1. We extended our analysis of sequence-specific effective solvation volumes to naturally occurring disordered linkers in multi-domain proteins within the non-redundant human proteome. Using a stringent set of criteria (see Materials and methods section) we identified approximately 100 linear multivalent proteins from the non-redundant human proteome (20,162 sequences) and extracted 226 unique linker regions (see Materials and methods for details). For each of the 226 linkers we performed all-atom simulations to quantify the sequence-specific values of ∆. The 226 sequences span a range of lengths (Figure 3c). We calculated the distribution of ∆ values for all linkers using results from all-atom simulations (Figure 3d). This distribution shows that sequences of naturally occurring disordered linkers span the entire range of ∆ values.

Of the 226 unique linker sequences, approximately 30% have negative effective solvation volumes (∆ < –0.1) whereas 38% have sequences defined by ∆ values in the range –0.1 ≤ ∆≤0.1, implying that they will have near zero effective solvation volumes and are mimics of FRC linkers. Finally, 30% of linkers are characterized by ∆ values greater than 0.1, which means that their effective solvation volumes are positive. The limiting form of a positive effective solvation volume linker is the self-avoiding random coil or SARC for which ∆ ≈ 0.5. The key finding is that disordered linkers come in a range of sequence flavors, and 68% have a positive or near positive effective solvation volume.

Supplementary file 1 summarizes key details regarding the naturally occurring linkers, including the protein name, UniProt identifier (Finn et al., 2014), the value of ∆, and Gene Ontology (GO) annotations. The linkers are derived from multivalent proteins associated with a range of different functions. The proteins we identified were significantly enriched for RNA/DNA binding and RNA localization, as assessed by PANTHER-GO enrichment analysis (Mi et al., 2017) (p<0.005). This is of particular relevance, given that many micron-sized biomolecular condensates contain protein and RNA molecules (Banani et al., 2017). With this analysis in hand, our next goal was to understand how different types of linkers might modulate the gelation transitions and overall phase behavior of linear multivalent proteins.

For linkers with negative effective solvation volumes the linkers serve as additional drivers of phase separation (Crick et al., 2013). These attractive linkers should be thought of as separate interaction domains and are hence distinct from regions that modulate the phase behavior of interaction domains. Therefore, we focused our studies on disordered linkers with near zero or positive effective solvation volumes (v_es_ ≥ 0).

### Design of coarse-grained simulations to model the phase behavior of linear multivalent proteins

Numerical simulations of phase transitions require the inclusion of hundreds to thousands of distinct multivalent proteins and a titration of a spectrum of protein concentrations. Furthermore, phase transitions are characterized by sharp changes to a small number of parameters, and the observation of these sharp transitions is computationally intractable with all-atom simulations. Therefore, we developed and deployed coarse-grained lattice models to study the impact of linkers on phase transitions. Parameters of the lattice models are summarized in Table 1 of the Materials and methods section.

Lattice models afford the advantage of a discretized conformational search space (Feric et al., 2016). This enables significant enhancements in computational efficiency. Key features of lattice models are the mapping of real protein architectures onto lattices and the design of an interaction model (Feric et al., 2016). The design of our simulation setup was inspired by the synthetic poly-SH3 and poly-PRM system studied by Li et al (Li et al., 2012). The general framework of our lattice model has been extended to other systems including branched multivalent proteins (Feric et al., 2016), and is transferable through phenomenological or machine learning approaches (Ruff et al., 2015) to any system of multivalent proteins and polynucleotides

**Figure 4.**
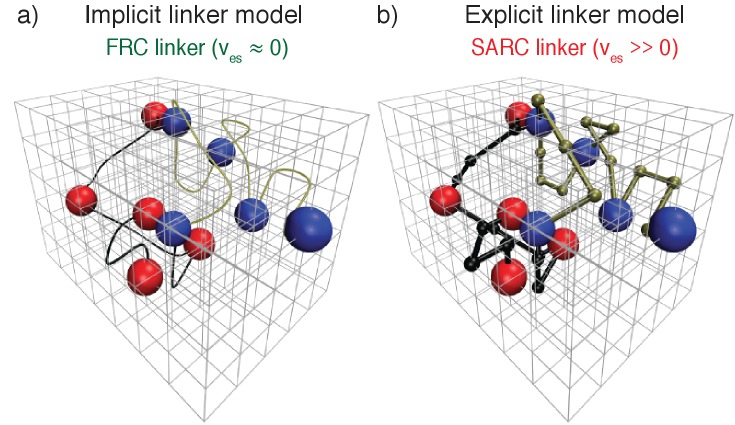
Coarse-grained bead-tether lattice models for modeling the phase behavior of multivalent proteins. All simulations were performed using 3-dimensional cubic lattice models. In these models, poly-SH3 and poly-PRM proteins were modeled as bead-tether polymers where the red beads mimic an SH3 domain, the blue beads mimic PRMs, and the black or gold tethers mimic linkers that connect domains/modules to one another. Two beads cannot occupy the same lattice site. Panel (**a**) shows an implicit linker model. To mimic FRC linkers, implicit linkers ensure that two tethered beads cannot move apart beyond a maximum distance, but the linker itself does not occupy any lattice sites. Panel (**b**) shows the explicit linker model. To mimic SARC linkers, explicit linkers consist of non-interacting beads corresponding to a prescribed number of lattice sites. The explicit linkers tether two folded domains together, but other than occupying sites on the lattice they do not engage in interactions with one another or with the interaction domains. Note that in the explicit linker model each linker bead and interaction domain occupies a single lattice site. This choice was motivated by previous analysis of the comparative effective solvation volumes of FRC and SARC linkers (Mittal et al., 2014). In the figure, the linker beads are represented as being smaller than the interaction beads to emphasize that they are linkers. The real simulation box used is much larger than the lattice dimensions pictured here, which is just for illustration purposes.

We modeled each multivalent poly-SH3 and poly-PRM protein using a coarse-grained bead-tether model (Figure 4). A single lattice site was assigned to each SH3 domain. This sets the fundamental length scale in our simulations. Each PRM comprises of approximately 10-residues, thus giving it the approximate dimensions of a single SH3 domain. Therefore, each PRM was also assigned to a single lattice site. Previous all-atom simulations showed that the spatial dimensions of a single SH3 domain corresponds to ~7 linker residues, if v_es_ ≥ 0 (54). Therefore, the linker length can be written as *N* ≈ 7 n where *n* is the number of lattice sites that span the linker and *N* is the number of linker residues. All simulations were performed on 3-dimensional cubic lattices with periodic boundary conditions. Individual SH3 domains and PRMs can bind to one another and form a 1:1 complex with an intrinsic binding energy of –2*k_B_T*. Here, *k_B_* is Boltzmann’s constant and *T* is temperature. This intrinsic affinity reproduces measured dissociation constants for SH3 domains and PRMs (Li et al., 2012).

We start with two stylized linkers namely, Flory random coil (FRC) linkers and the self-avoiding random coil (SARC) linkers. FRC linkers correspond to chains with v_es_ = 0. We model FRC linkers as implicit linkers (Figure 3a) – the linkers have a fixed length and tether the domains together, but do not occupy any volume on the lattice. Practically this is realized by imposing a cubic infinite square well potential to ensure that the lattice spacing between tethered interaction domains does not exceed *n,* which is the linker length in terms of the number of lattice sites. For the SARC linkers with positive v_es_, we use explicit linkers as shown in Figure 3b. A SARC linker of length *n* has *n* beads, where each bead is constrained to occupy vertices adjacent to its nearest neighbor beads on the lattice. Each explicitly modeled linker bead occupies a finite volume corresponding to one lattice site.

### Parameters to distinguish between phase separation and gelation

**Figure 5.**
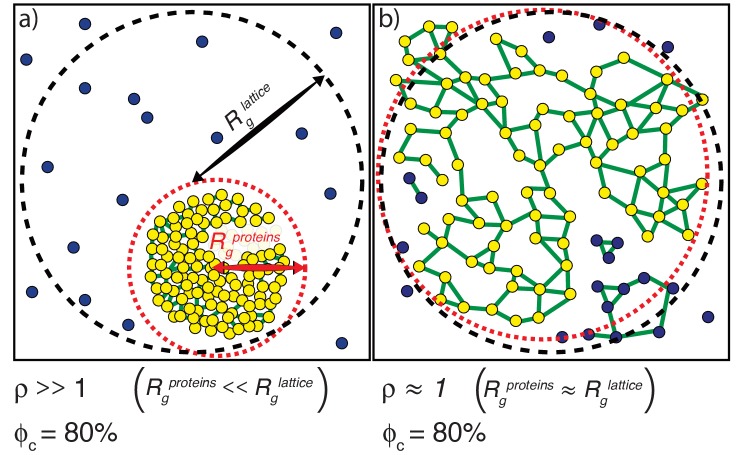
Illustration of how ρand ϕ_c_ are calculated. (**a**) *The scenario where ρ >>1.* The radius of gyration over all proteins is the root mean square distance of each of the proteins from the center of mass of the system of proteins and is depicted as the radius of the dashed red envelope. Although the red envelope is centered on the cluster, it extends beyond the cluster boundary due to the presence of proteins outside of the cluster; that is, *R_g_^proteins^* is always calculated over *all* proteins in the system. When a majority of the proteins are spatially clustered, the calculated *R_g_^proteins^* is considerably smaller than the radius of the lattice, and hence the ratio ρ >>1. *R_g_^lattice^* is shown as a black dashed envelope. In panel (**a**) a majority of the proteins are found within a single droplet-spanning cluster. This cluster encompasses ~ 80% of the modules, hence ϕ_c_ ~80%. Modules belonging to the single largest system spanning clusters are shown in yellow, the crosslinks are shown in green, and the ‘system’ here refers to the droplet. (**b**) *The scenario where ρ ≈ 1*. In this case, the modules are dispersed across the lattice volume as shown by the fact that the dashed red envelope is essentially coincident with the dashed black envelope. Here, we depict a scenario where 80% of the modules are incorporated into the single largest system-spanning cluster, where the ‘system’ volume corresponds to that of the entire lattice.

Phase separation results from a change in density. We quantify a parameter ρ, which we define as the ratio of *R*_lattice_ to $R_{g}^{proteins}$. Here, *R*_lattice_ is the radius that we would obtain if all proteins were uniformly dispersed across the lattice (Figure 5). Conversely, $R_{g}^{proteins}$is the actual ensemble-averaged radius of gyration over the spatial dimensions of the SH3, PRM, and linker beads (Figure 5). For a system that has undergone phase separation, the parameter ρ will be >1. ρ is directly related to the relative density of the proteins and measures the extent of spatial clustering of domains and linker residues. If ρ is equal to one, then the proteins are uniformly dispersed through the lattice.

We quantify gelation in terms of the fraction of molecules in the system that are part the single largest cluster. This is denoted as ϕ_c_ (Figure 5). We analyze each configuration of multivalent proteins to detect the formation of connected clusters. Within each configuration, each molecule is a *node*. An *edge* is drawn between two *nodes* if an SH3 domain from one molecule interacts with a PRM from another molecule. The connected cluster with the largest number of nodes is designated as the largest cluster and the number of molecules corresponding to this cluster is recorded. This quantity is calculated across the entire ensemble of configurations in order to generate an ensemble averaged value of ϕ_c_ for the system of interest. As a result of the finite surface tension associated with droplet formation and the precautions taken to reach convergence (see Materials and methods), we find that the single largest cluster absorbs all other clusters, thus giving rise to a true two-phase system as pictured in Figure 5a.

### Multivalent proteins with FRC linkers undergo gelation driven by phase separation

We performed a series of Monte Carlo simulations using a coarse-grained lattice model for poly-SH3 and poly-PRM systems of valence 3, 5, and 7 and all combinations of these valencies. Unless otherwise specified, in all of our simulations, the linker length *n* was set to five lattice sites, approximately 35 residues. This linker length corresponds to the main mode in the distribution of linker lengths shown in Figure 3c.

**Figure 6.**
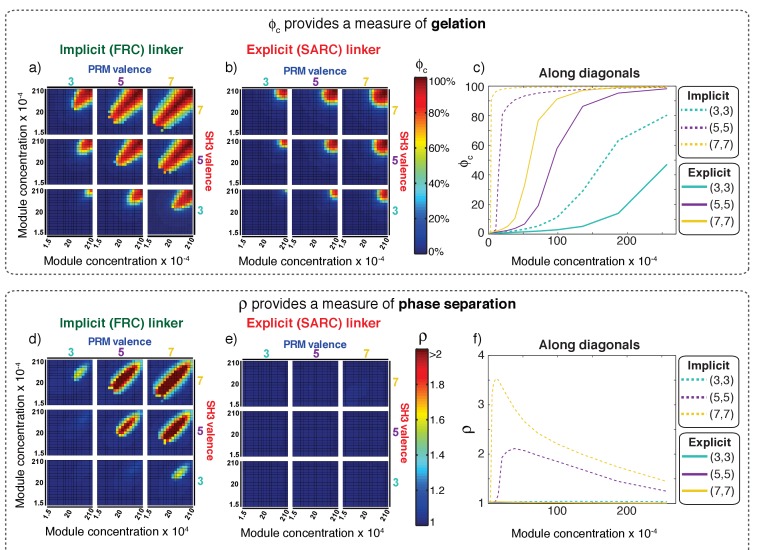
Comparative analysis of the connectivity and density transitions for multivalent proteins of fixed linker lengths. (**a**) Heat maps showing ϕ_c_ as a function of changes to SH3 and PRM concentrations for multivalent proteins with FRC linkers. Progression from cool to hot colors leads to the incorporation of most of the modules into the single largest cluster. The module concentrations at which sharp changes in connectivity are realized will decrease with increasing valence. (**b**) Heat maps equivalent to those of panel (**a**) for multivalent proteins with SARC linkers. (**c**) Analysis of how ϕ_c_ changes with module concentration for equal concentrations SH3 modules to PRMs. The solid curves plot ϕ_c_ for proteins with SARC linkers and the dashed curves are results for FRC linkers. The legend provides an annotation of the color scheme for the different curves. (**d**) Heat maps showing ρ as a function of changes to SH3 and PRM concentrations for multivalent proteins with FRC linkers. Comparison to panel (**a**) shows the congruence between changes to ρ and ϕ_c_, especially for the 5:5, 5:7, 7:5, and 7:7 systems. (**e**) Heat maps showing ρ as a function of changes to SH3 and PRM concentrations for multivalent proteins with SARC linkers. The value of ρ does not change and remains close to one irrespective of the valence or module concentration. (**f**) Analysis of how ρ changes with module concentration for equal concentrations SH3 modules to PRMs. The solid curves are for proteins with SARC linkers and this shows that ρ ≈ 1, irrespective of the module concentrations. As discussed in the text and summarized in Figure 7, phase separation is suppressed for systems with SARC linkers and this is reflected in the invariance of ρ. The dashed curves, for the 5:5 and 7:7 systems with FRC linkers show a sharp change above a threshold concentration of the modules. The behavior at high module concentrations is partly an artifact of our approach to increasing concentrations in the simulations, which involves fixing the number of modules and decreasing the volume of the simulation box. Accordingly, the radius of the lattice will decrease, thus decreasing ρ. However, ρ is greater than one above a critical concentration, thus emphasizing the coupling between phase separation and gelation for proteins with FRC linkers.

The first row of plots in Figure 6 shows how ϕ_c_ changes for different simulated systems and provides a quantification of gelation. Each sub-plot in Figure 6a shows the value of ϕ_c_ as a function of the concentrations of SH3 domains and PRMs for a particular combination of PRM and SH3 domain valence. Figure 6a establishes two distinctive features of multivalent systems: For a given combination of SH3 and PRM valencies, we observe a sharp increase in the values of ϕ_c_ as the concentrations of SH3 domains and PRMs increase. This behavior is consistent with the expected features of a sol-gel transition. Second, as valence increases, there is a lowering of the module concentrations at which ϕ_c_ increases sharply.

Figure 6b shows results for ϕ_c_ obtained for poly-SH3 and poly-PRM systems with SARC linkers. Here, five beads were modeled explicitly for each of the linkers between SH3 domains and PRMs. Although most systems show a sharp increase in ϕ_c_ past a threshold SH3/PRM concentration, the concentrations at which the transitions are realized are at least an order of magnitude higher than those observed for the systems with FRC linkers. The differences between FRC and SARC linkers are summarized in Figure 6c, which shows how ϕ_c_ changes with module concentrations for the symmetric 3:3, 5:5, and 7:7 systems along the diagonals for equal ratios of SH3 domains and PRMs. The *x: y* designation refers to the *valence of SH3 domains: the valence of PRMs*. The value of ϕ_c_ changes sharply with concentration and this change becomes sharper as the valence increases. For a given valence, ϕ_c_ increases more sharply and this sharp change happens at lower module concentrations for proteins with FRC as opposed to SARC linkers. This analysis shows that the effective solvation volumes of linkers can have a profound impact on sol-gel transitions.

The bottom row in Figure 6 shows how ρ changes for each of the multivalent systems and provides a quantification of phase separation. Figure 6d, which summarizes the results for FRC linkers, shows sharp changes to ρ as valence increases. This recapitulates the observations in Figure 6a for ϕ_c_ indicating that changes to connectivity are coupled to changes in density. This is illustrated in plots for the 7:7, 7:5, 5:7, and 5:5 systems. In contrast, the 5:3, 3:5, and 3:3 systems show gelation transitions with negligible changes to ρ. In the highly asymmetric 7:3 and 3:7 systems, the changes in ρ are considerably less pronounced when compared to changes in ϕ_c_. In each simulation, the initial conditions correspond to the multivalent proteins being randomly dispersed across the cubic lattice (see Video 1). The movie and comparative analysis of results in Figure 6a and d provide visual support for the suggestion that systems with FRC linkers undergo phase separation plus gelation.

**Video 1.** Demonstration of gelation driven by phase separation for the 7:7 system of poly-SH3 and poly-PRM. The color-coding is such that SH3 domains are in red and PRMs are in blue. The simulations start with the molecules dispersed uniformly across the simulation volume. The movie shows droplet formation leading to gelation for bulk concentrations of SH3 domains and PRMs that lie above the saturation concentration *c_sl_*.

Figure 6e shows the results obtained for poly-SH3 and poly-PRM systems with SARC linkers. The results provide a striking contrast to the results obtained for proteins with FRC linkers (see Video 2 ). None of the systems show discernible changes to ρ. This implies that gelation occurs only when the concentrations are large enough to enable networking through random encounters. The positive effective solvation volumes of SARC linkers suppress phase separation and these systems undergo gelation without phase separation. Figure 6f summarizes the distinctions between FRC and SARC linkers by plotting ρ versus the concentration of modules for the symmetric cases with equal ratios of SH3 domains and PRMs. For SARC linkers, ρ ≈ 1 across the entire concentration range for (solid curves). This emphasizes the suppression of phase separation for systems with SARC linkers. For proteins with FRC linkers, the values of ρ increase sharply above unity beyond system-specific critical concentrations.

**Video 2.** Demonstration of gelation without phase separation for the 7:7 system of poly-SH3 and poly-PRM. The movie shows the formation of a system-spanning network formation leading to gelation for bulk concentrations of SH3 domains and PRMs that lie above the gel point *c_g_*.

**Figure 7.**
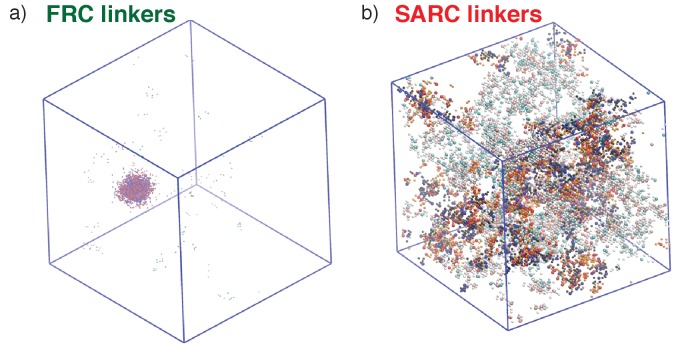
Representative, post-equilibration, snapshots for the 7:7 system above the gel points with FRC, panel (**a**), and SARC linkers, panel (**b**) of length *n* = 5. In panel (**a**), the SH3 modules are shown in red and the PRMs in blue. In panel (**b**), the coloring is similar to panel (**a**). Additionally, molecules that are part of the single largest, system-spanning cluster are shown in orange. The main message conveyed here is that the SARC linkers suppress phase separation whereas the FRC linkers lead to gelation driven by phase separation.

Representative post-equilibration configurations for 7:7 systems with FRC and SARC linkers of length five are shown in Figure 7. Both snapshots correspond to values of ϕ_c_ being above the gel point. The bounding box corresponds to the volume of the simulation cell and provides perspective regarding the change in density and networking within the system. In Figure 7a,a dense (high ρ) spherical droplet, which is a gel (ϕ_c_ is above the percolation threshold), coexists with a dilute sol of well-dispersed proteins. In contrast, Figure 7b shows how a system spanning network, that is, gelation occurs in the absence of phase separation.

### Linkers influence the degree and type of cooperativity in sol-gel transitions

If the linkers are short, then irrespective of the effective solvation volume, the formation of a physical crosslink between a pair of multivalent proteins will increase the probability that a second crosslink can form between the same pair of proteins. In this scenario, there is *positive local cooperativity,* in that the apparent affinities will increase (Jencks, 1981) but the network cannot grow because the apparent valence is lower than the actual valence. In the limit of positive local cooperativity, phase separation and gelation are suppressed because collective interactions amongst the molecules are weakened in favor of forming network terminating dimers and oligomers. This scenario corresponds to *infinite negative global cooperativity*. In this scenario, there will neither be gelation nor phase separation.

It the linkers go beyond a system-specific length, the domains will become independent of one another. Here, the extent of crosslinking and the gel point are determined entirely by the valence of domains and the intrinsic affinities between domains. This is the limit of classical Flory-Stockmayer theories (Flory, 1941; Flory, 1942b; Stockmayer, 1943) with *zero local cooperativity*. The linkers are passive tethers that generate multivalency, but they do not make any other contributions to the transitions of multivalent systems. In the limit of zero local cooperativity, gelation occurs without phase separation and the apparent valence equals the actual number of domains, implying *zero global cooperativity*.

For intermediate linker lengths, the signs and magnitudes of the effective solvation volumes of linkers will determine the overall phase behavior. Disordered linkers with negative or near zero v_es_ values can enable phase transitions characterized by *positive global cooperativity* because they can drive density transitions of multivalent proteins. These linkers can be confined to small volumes, when compared to the volume of the entire system. This derives from the preference for chain-chain interactions (v_es_ < 0) or indifference for chain-chain versus chain-solvent interactions (v_es_ ≈ 0). Increased concentrations of domains within confined volumes realized by density transitions will enable networking transitions because the gel point is now lower than the concentration of domains within the dense phase. If a multivalent protein contributes to growth of a network by forming a crosslink with a free domain on a protein that has already formed a crosslink with another protein, then the increased crosslinking enables gelation. These collective effects can also increase the apparent affinities between domains (as in the first scenario) thereby increasing the concentration of interaction domains. Increased crosslinking enables a networking transition whereas increased concentration of domains enables a density transition. The regime of positive global cooperativity corresponds to the regime where gelation is driven by phase separation.

Linear multivalent proteins with large positive effective solvation volume linkers (v_es_ >> 0) will engender *negative global cooperativity* because the linkers prefer to be solvated and will resist confinement within droplets. In this sense, linkers with large positive effective solvation volumes are analogous to solubilizing tags. Additionally, due to their large positive effective solvation volumes, the linkers act as obstacles that inhibit interactions between domains. These linkers decrease the apparent affinity between interaction domains and reduce the degree of crosslinking. Accordingly, the ability to concentrate multivalent proteins is weakened, and so is the ability to grow a system-spanning network via a connectivity transition. In the scenario of negative global cooperativity, phase separation is suppressed and gelation is realized at bulk concentrations that are considerably higher than the Flory-Stockmayer limit. As a reminder, linkers do not make any contribution to determining the gel point in the Flory-Stockmayer limit (Flory, 1974, 1941, 1942b; Stockmayer, 1943), only the valence and intrinsic affinities matter.

To summarize, gelation driven by phase separation will lead to positive global cooperativity, and enable the formation of a percolated network at bulk concentrations that are considerably smaller than the Flory-Stockmayer limit. Systems with zero or negative global cooperativity undergo gelation without phase separation and sol-gel transitions occur at or above the Flory-Stockmayer limit.

### A dimensionless parameter to quantify cooperativity

To put the ideas described above on a quantitative footing and enable comparisons across different systems we calculated the percolation threshold in terms of ϕ_c_, and we designate this as ϕ_cc_. We then use the value of ϕ_cc_ to quantify the gel point *c_g_*. The gel point is the concentration threshold beyond which the system crosses the percolation threshold. The methods for computing ϕ_cc_ for a system with prescribed values for the valence and the binding energy between interaction domains, as well as the calculation of the gel point from ϕ_cc,_ are described in the methods section.

We introduced a dimensionless parameter c* to quantify the magnitude and type of cooperativity that characterizes phase transitions of linear multivalent proteins. The parameter c* is defined as the ratio of *c_g_*_,sim_ to *c_g_*_,FS_, that is, c* = (*c_g_*_,sim_/*c_g_*_,FS_). Here, *c_g_*_,sim_ is the gel point quantified in simulations with linkers of specified length and effective solvation volume. It is defined as the lowest concentration of modules at which **ϕ_c_**>0.17. This is the percolation threshold for our system of finite-sized linear multivalent proteins (see Materials and methods section). In contrast, *c_g_*_,FS_ is the gel point obtained from Flory-Stockmayer theories (Flory, 1974, 1941, 1942b; Stockmayer, 1943). Therefore, the value of *c_g_*_,FS_ provides an important touchstone for quantifying the influence of linkers on phase transitions, and provides a measure of the deviation from the mean-field behavior expected of long inert linkers. For a system with positive global cooperativity, c*<1; for a system with zero global cooperativity, c*=1; and for a system with negative global cooperativity, c*>1. The value of c* quantifies the joint effects on changes to the apparent affinities of interaction modules and the extent of crosslinking.

### FRC linkers have an optimal range of lengths for positive cooperativity

**Figure 8.**
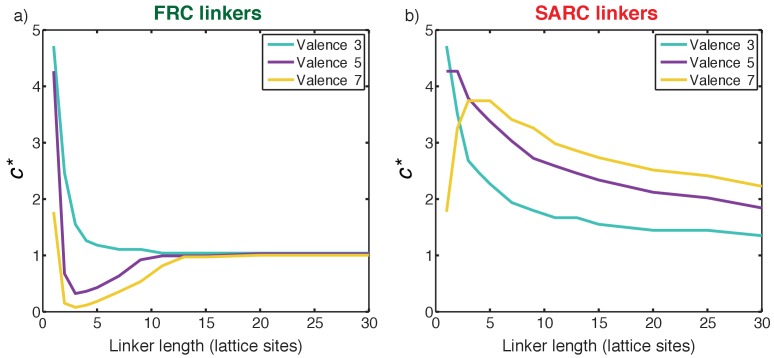
Quantifying cooperativity and the coupling between phase separation and gelation. (**a**) Plot of c* as a function of linker length for three symmetric multivalent systems connected by FRC linkers. There is an optimal range for linker lengths where c*<1, implying positive global cooperativity that gives rise to phase separation plus gelation. For long linkers, c* converges to unity, implying an absence of cooperativity and pure sol-gel transitions, in accord with Flory-Stockmayer theories. (**b**) Plot of c* as a function of linker length for three symmetric multivalent systems connected by SARC linkers. The value of c* is greater than unity for all linker lengths. This points to the suppression of phase separation by linkers with positive effective solvation volumes, and a shifting of the gel point to higher concentrations compared to the Flory-Stockmayer threshold. The linker length in terms of number of amino acids can be written as *N* ≈ 7 n, where *n* is the number of lattice sites and *N* is the number of residues.

We quantified the impact of linker lengths on the degree and magnitude of cooperativity for FRC linkers. Figure 8a shows a plot of c* as a function of linker lengths for 3:3, 5:5, and 7:7 systems with FRC linkers. The profile of c* is non-monotonic. In the short linker limit (*n* ≤ 2) the value of c* is greater than one. These linkers are too short and therefore complexes terminate in dimers of poly-SH3 and poly-PRM proteins. This is the regime of positive local and negative global cooperativity where phase transitions do not occur.

For multivalent proteins with a valance of 5 or 7 and linker lengths in the range 3 ≤ *n* < 12 (or 21 ≤ *N* ≤ 84, where *N* is the number of linker residues), the value of c* is less than one, and the lowest values of c* are realized for linkers of length 3 < *n* < 6. FRC linkers within a defined length range engender positive global cooperativity and for linker lengths in this optimal range, positive global cooperativity increases with increasing valence. This is the regime where phase separation promotes gelation and c* is less than 1. Positive global cooperativity weakens with increasing linker lengths. Accordingly, for long linker lengths, c* converges to one implying that the domains interact independently when the FRC linkers are sufficiently long. This is the regime of zero global cooperativity where gelation occurs without phase separation in accord with the predictions of Flory-Stockmayer theory (Stockmayer, 1943).

### SARC linkers lead to negative global cooperativity

Figure 8b shows a plot of c* as a function of linker lengths for 3:3, 5:5, and 7:7 systems with SARC linkers. Here, c* is greater than one for all the linker lengths. This is a signature of negative global cooperativity. Linkers with positive effective solvation volumes suppress phase separation and shift the gel point to higher concentrations when compared to the threshold predicted by Flory-Stockmayer theories. Explicit linkers also lower the apparent affinity through negative global cooperativity because their positive effective solvation volumes promote solvation thus diminishing productive associations among domains. This becomes less of an issue as the linkers become longer. If one corrects the intrinsic affinity to account for the weakened apparent affinity, then the convergence of the systems with long linkers to the Flory-Stockmayer limit is recovered (not shown). However, the profiles do not change qualitatively and this points to fundamental differences between systems with FRC versus SARC linkers.

The analysis in Figure 8 has ramifications for drawing inferences from the proteome-wide analysis summarized in Figure 3. We find that the values of ∆ and linker length are essentially uncorrelated. This is not surprising because the main determinant of the effective solvation volumes is the sequence/amino acid composition and not the length of the linker. This point is underscored in the analysis summarized in Figure 3. Our analysis of linker sequences in linear multivalent systems shows that approximately 30% of all linkers in the inventory will have 50 or fewer residues and ∆ values less than 0.1 (Supplementary file 1). These linkers are the most likely candidates for enabling gelation driven by phase separation in linear multivalent proteins. Approximately, 18% of all linkers have fewer than 50 residues and ∆ values greater than 0.1. These are the most likely candidates for weakening phase separation and sequences with large positive values of ∆ will drive gelation without phase separation. The remainder of the linkers,~50% in all, are longer than 50 residues and these are unlikely to be major modulators of gelation transitions since the analysis in Figure 8 suggests that these linkers cross into the Flory-Stockmayer limit, where the interaction modules become independent of one another.

### Phase diagrams delineate parameters for distinct types of phase transitions

**Figure 9.**
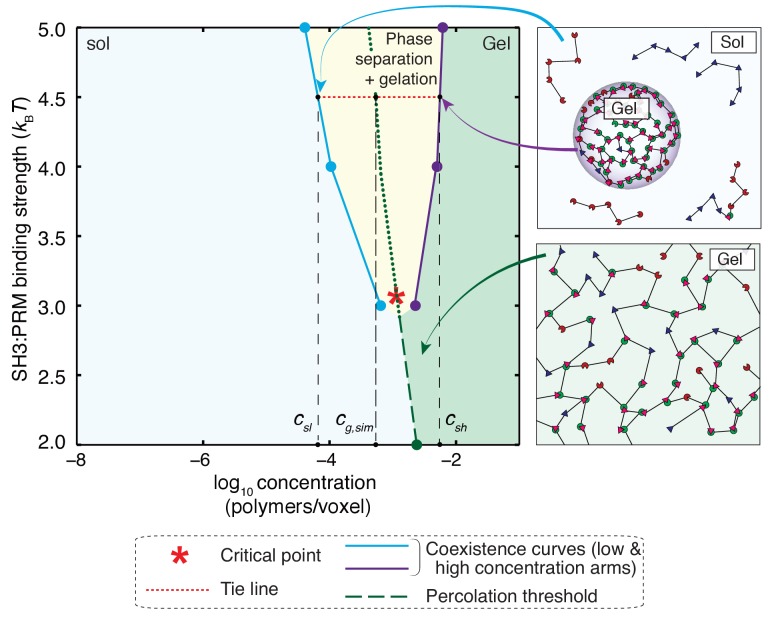
Phase diagram for a 5:5 system with a hybrid five-site linker. Here, for each linker, two of the linker beads were modeled explicitly, while the other three were modeled implicitly. For low binding affinities between SH3 domains and PRMs (<3 *_k_BT*), the system undergoes a sol-gel transition as a function of module concentration, and the affinity-specific gel points lie on the green dashed line. The red asterisk denotes the critical point located at an interaction affinity of ~3 *_k_BT* and a module concentration of ~10^–3^polymers/voxel. Above an interaction affinity of ~3 *_k_BT*, the system undergoes phase separation plus gelation. Phase separation is characterized by a coexistence curve with two arms, shown in blue and purple. A solution with a bulk concentration that falls within the yellow region will never form a one-phase solution. Instead, it will separate into coexisting dilute and dense phases. The concentrations within these phases are equal to the concentrations taken from coexistence curves that intersect with the corresponding tie line (red dotted line). This is illustrated for interaction strengths of 4.5*k_B_T*. Any solution with a bulk concentration along the tie line will phase separate into a dense phase and a dilute phase of a fixed concentration *c_sl_* and *c_sh_*, respectively. For this system, the high concentration arm of the coexistence curve always lies beyond the gel-line, and therefore, the dense phase will always form a gel. The gel line within the two-phase region is calculated based on the percolation threshold and is shown as a dotted green line, which is really an extrapolation of the green dashed line. It highlights the fact that *c_sl_* <*c_g_ < *_csh_ throughout the two-phase regime. The callouts on the right show schematics of the dilute sol coexisting with a dense gel (top right) and a system spanning gel that forms via gelation without phase separation (bottom right).

Figure 9 shows the phase diagram that we computed from concentration dependent simulations for a 5:5 system and a hybrid five-site linker. This phase diagram is shown in the two-parameter space of the concentration of domains along the abscissa and increasing intrinsic affinities along the ordinate. For affinities below 3*k_B_T*, the system undergoes a continuous transition from a sol to a gel and the green dashed line demarcates the sol-gel line. The gels correspond to system-spanning networks that percolate through the entire simulation volume. The critical point for this system, shown as a red asterisk, is defined jointly by a critical interaction affinity (3*k_B_T*) and a critical module concentration (~10^–3^polymers/voxel).

Above the critical point, the system undergoes gelation driven by phase separation. As the interaction affinity increases above 3*k_B_T*, the system separates into two coexisting phases namely, a dilute phase, which is a sol, and a dense phase, which is a gel. As an illustration, for an interaction affinity of 4.5*k_B_T*, the coexisting concentrations that define the two phases are designated as *c_sl_* and *c_sh_*, which are respectively the concentrations of dilute and dense phases. Notice that the gel point, *c*_g_, defined as the concentration beyond the percolation threshold, ϕ_c_ > 0.17, lies within the two-phase regime such that *c_sl_* < *c_g_* < *c_sh_*. Here, *c_g_* is the apparent gel point that is extrapolated by extending the green dashed line in Figure 9. Accordingly, the density transition, which we quantify as the concentration range above which ρ becomes greater than 1.08, enables gelation because the concentration within the dense phase (*c_sh_*) is higher than the apparent gel point (*c_g_*). The result is a droplet-spanning network as pictured in the Figure 7a.

The width of the two-phase regime increases with interaction affinity. This implies that phase separation is realized at lower concentrations of the interacting domains and is depicted by a leftward shift of the arm shown in light blue in Figure 9. Concomitantly the droplet becomes more concentrated and this is depicted by a rightward shift of the arm shown in purple in Figure 9. Therefore, if the linker sequence is fixed, mutations to interaction domains or SLiMs that increase affinity will enhance phase separation, giving rise to concentrated droplets encompassing gels that coexist with dilute sols.

### Phase separation is destabilized as the effective solvation volumes of linkers increase

**Figure 10.**
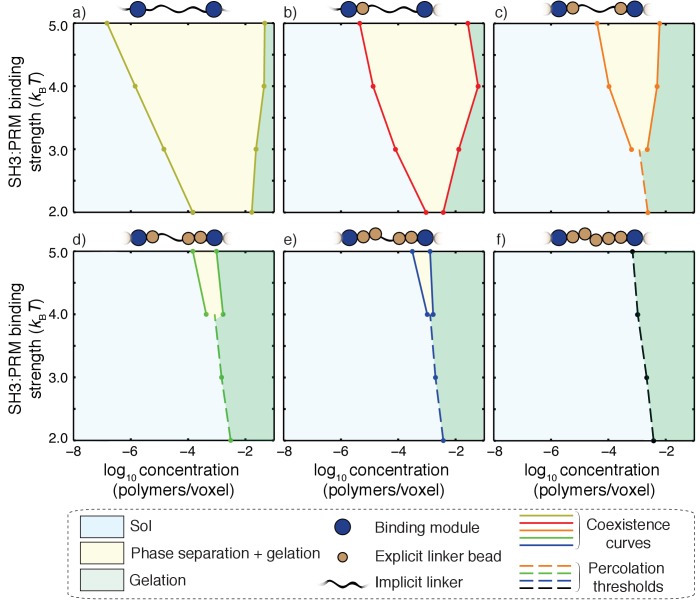
Impact of linker v_es_ values on coupling between phase separation and gelation for 5:5 systems with linkers of length *n* = 5. Progressing from panel a) to panel f), the value of v_es_ for each of the linkers increases from 0 to 5 in terms of number of lattice units. The widths of the regimes that correspond to phase separation (yellow regions) shrink as the effective solvation volumes of linkers increase. For the fully implicit, FRC linker (panel a), gelation without phase separation either requires shorter linkers or interaction affinities that are weaker than 2*k_B_T*. The sol-gel lines are shown as dashed lines in each panel. Accordingly, for a) and b) the gelation without phase separation are realized for SH3: PRM affinities that are weaker than 2*k_B_T* and hence they are not shown in these panels. Each panel is annotated with a schematic to show the design of hybrid linkers and each schematic we shown only a single linker for clarity.

The effective solvation volumes of linkers were titrated by fixing the linker length and changing the number of linker beads that were modeled implicitly versus explicitly. The magnitude of the effective solvation volume is quantified in terms of the number of explicitly modeled beads within each linker. For example, if two out of five linker beads are modeled explicitly, then v_es_ is proportional to the volume of two lattice units as is the case for linkers that yield phase diagrams shown in Figures 9 and 10c.

Each of the panels in Figure 10 corresponds to a distinct type of linker, defined by the effective solvation volume, that is, the number of explicitly modeled linker beads for a linker of length five. The results are shown for interaction affinities of modules that range from 2*k_B_T* to 5*k_B_T*. Progressing from the top left corner to the bottom right corner, we find that the critical point shifts to higher interaction affinities as the effective solvation volumes of linkers increase. If the linkers have more of an FRC-like character, then the phase transitions are likely to fit the description of being gelation driven by phase separation. For a given value of the affinity, the width of the two-phase regime increases as the magnitude of the effective solvation volume decreases. In contrast, the two-phase regime becomes negligibly small as the magnitude of the linker effective solvation volume increases. In fact, for high effective solvation volumes of linkers, the presence of a two-phase regime is discernible only for very high affinities and phase transitions occur mainly via gelation without phase separation.

## Discussion

Using numerical simulations, we show that that linear multivalent proteins can undergo two distinct types of transitions namely, *gelation without phase separation* and *gelation driven by phase separation*. We also showed that linkers between domains/motifs in linear multivalent proteins are not just passive tethers. In addition to serving as scaffolds for motifs (Das et al., 2016; Banjade et al., 2015), the physical properties of linkers such as their lengths and effective solvation volumes will directly influence the extent to which phase separation promotes gelation (Semenov and Rubinstein, 1998).

The distinction between gelation without phase separation and gelation driven by phase separation was formalized in the theoretical work of Semenov and Rubinstein (Semenov and Rubinstein, 1998; Rubinstein and Semenov, 1998). In their mean-field model for infinitely long associative polymers, phase separation facilitates gelation for chains with negative, near zero, or mildly positive effective solvation volumes. Phase separation is suppressed as v_es_ becomes positive and extent to which phase separation promotes gelation is modulated by the affinities between associative domains/motifs. Our numerical results summarized in Figures 6–10 are consistent with the theoretical predictions of Semenov and Rubinstein. This is gratifying given that we focus on finite-sized polymers where the simplifications of mean field theories are not necessarily transferrable. We have also shown that the effective solvation volumes of linkers are directly determined by their primary sequences (Figure 3). Finally, there appears to be an optimal range of linker lengths that supports gelation driven by phase separation for a given interaction affinity between domains.

We focused our simulations of phase transitions on linkers with zero or positive v_es_ values. However, as shown in Figure 3d, approximately 30% of linkers in the sub-proteome of linear multivalent proteins have negative v_es_ values. These linkers will be self-attractive. They can also engage in non-specific attractive interactions with interaction domains as well as other linkers of different sequence composition that have negative v_es_ values. Linkers with negative v_es_ values are best thought of as additional interaction sites. Therefore, linkers with negative v_es_ values have two distinct effects: firstly, they lead to be an effective shortening of the linker length due to linker compaction, and secondly they can engage in additional in trans interactions causing an increase in the effective valence. These effects were illustrated in a previous study that was designed to study coexisting dense phases formed by the intrinsically disordered RGG domain of the protein Fibrillarin-1 (FIB1). There, the RGG domain of FIB1 was modeled using five explicit sticky beads thus conferring an effectively negative v_es_ value on this domain (Feric et al., 2016). Linkers with negative v_es_ values are likely to yield significantly more dense droplets when compared to linkers with near zero or positive v_es_ values. This is underscored in recent measurements of intra-droplet concentrations for disordered proteins with positive (Wei et al., 2017) versus negative v_es_ values (Simon et al., 2017). The intra-droplet concentration for the RGG domain of LAF-1 (41), which has a positive v_es_ value, is two orders of magnitude smaller than the intra-droplet concentration measured for elastin-like polypeptides (Simon et al., 2017), which have negative v_es_ values.

Interestingly, the sequences of many low complexity domains that tether RNA recognition modules in proteins such as hnRNP-A1 and FUS have negative v_es_ values. The high density within these droplets might explain why disease-associated mutations within these sequences engender apparently pathological gelation transitions that appear to be aided by conformational changes into beta-sheet-rich fibrils (Lee et al., 2016; Molliex et al., 2015; Patel et al., 2015; Burke et al., 2015; Conicella et al., 2016; Weber and Brangwynne, 2012). In contrast, linkers characterized by mildly negative, zero, or mildly positive v_es_ values might form reasonably dilute droplets and functional gels that suppress pathological transitions (Li et al., 2012; Banjade and Rosen, 2014; Riback et al., 2017; Banani et al., 2016; Banjade et al., 2015).

Linear multivalent proteins are associative polymers, will undergo gelation with or without phase separation. We speculate that the regulation of cell signaling by phase transitions might predominantly involve gelation driven by phase separation. This is evidenced by the formation of spherical droplets that is driven by specific multivalent proteins comprising of multiple interaction domains or linear motifs (Su et al., 2016; Li et al., 2012; Banjade and Rosen, 2014; Banani et al., 2016; Jiang et al., 2015; Bergeron-Sandoval et al., 2016). The role of phase separation in cell signaling likely reflects the fact that the formation of dense droplets will increase local protein concentrations, facilitating a cooperative amplification in signal transduction as distinct signaling components undergo efficient intermolecular phosphorylation due to the high local concentration (Su et al., 2016; Hernández-Vega et al., 2017; Woodruff et al., 2017). Sequestration of key proteins into compartments also seems to be an important biological function that is achievable via phase separation (Shin and Brangwynne, 2017; Riback et al., 2017). Gelation within a droplet will contribute directly to the droplet sub-structure and to the spatial organization of components within droplets (Li et al., 2012). The extent and dynamics of crosslinking within a droplet-spanning gel will directly influence the material properties of droplets (Tanaka, 2011). These properties include the void volumes, average mesh sizes, local stiffness, dimensionality of the confined space, and rheological properties such as the viscoelastic profiles of membraneless bodies (Tanaka, 2011; Semenov and Rubinstein, 1998; Rubinstein and Colby, 2003; Rubinstein and Semenov, 1998). A striking example of the functional relevance of gelation was recently reported for S-crystallin proteins that make up the refractive material of the squid lens (Cai et al., 2017). The physics of phase separation is insufficient to explain the formation of a gradient of protein volume fractions across the lens. However, the measured features of the squid lens are readily explained using the framework of patchy colloid theory (Bianchi et al., 2006; Bianchi et al., 2008), whereby the polydispersity of disordered loops in S-crystallin determine the extent of physical crosslinking giving rise to gels of different densities across the lens (Cai et al., 2017).

Gelation without phase separation may also be useful in biology. Halfmann has reviewed functional scenarios where low complexity domains might undergo dynamical glass transitions that can resemble gelation without phase separation (Halfmann, 2016). The glass transitions of the inactive bacterial cytosol and the transition to ‘solid-like’ materials in fungi as a response to pH induced stresses are examples of sol-gel transitions on the whole cell level that do not have the characteristic hallmarks of accompanying phase separation of specific components (Parry et al., 2014; Munder et al., 2016).

Phase separation without gelation requires that the concentration within the dense phase be lower than the gel point (*c_sl_* < *c_sh_* < *c_g_*). For associative polymers, given the hierarchy of specific interactions that are encoded by the domains/motifs, it is difficult to envisage a scenario where the interactions would be strong enough to drive phase separation without the formation of physical crosslinks. While our work does not explore the dynamics associated with gelation, there are various lines of evidence that under certain scenarios the liquid-to-solid transition observed within droplet may by refectory for biological function (Patel et al., 2015; Mateju et al., 2017). If the formation of gels with solid-like properties is deleterious, then it is likely that active processes within the cell inhibit this transition within dense droplets, such that physical crosslinks are actively sheared (Mateju et al., 2017). Such a scenario would be an example of a so-called active liquid (Protter and Parker, 2016; Brangwynne et al., 2011) or more precisely a *non-equilibrium liquid* where energy is expended to suppress or limit gelation that would accompany phase separation of multivalent proteins (Brangwynne et al., 2015). Competitor molecules such as specific RNA sequences might also enable a shearing of percolated networks (Lee et al., 2015), although this has not been formally proven.

We further propose that effective scaffolding proteins for gelation driven by phase separation are likely to be linear multivalent proteins with linkers that have low effective solvation volumes (v_es_ ≈ 0). Proteins with linkers that have large positive v_es_ values are likely to be clients that partition into the droplets formed by the scaffolds (Banani et al., 2017). The precise nature of phase transitions might be biologically tunable. For example, the effective solvation volumes of linkers in linear multivalent protein can be tuned through synergistic actions of kinases and phosphatases (Bergeron-Sandoval et al., 2016; Kwon et al., 2013). This will alter the fraction of charged residues along linkers thus enabling an alteration of the phase behavior by altering the effective solvation volumes of linkers. Support for this proposal comes from the observation that the substrates for multisite phosphorylation tend to be enriched in disordered regions with positive effective solvation volumes (Holehouse et al., 2017; Martin et al., 2016). Additionally, posttranscriptional processing of mRNA transcripts via alternative splicing can also be a route for making tissue-specific alterations to linker sequences. Interestingly, transcripts coding for disordered regions are preferentially targeted by tissue-specific splice factors when compared to transcripts for folded domains (Buljan et al., 2013; Buljan et al., 2012).

The inventory of linker sequences, shown in Supplementary file 1, combined with the analysis presented in our numerical simulations, provides a ready-made route to search for candidate linear multivalent proteins that drive gelation driven by phase separation plus gelation versus gelation without phase separation. Clearly, we need detailed experimental and theoretical characterization of phase diagrams of multivalent proteins, with special attention to the intersection of sol-gel lines and the two-phase regime (Figures 9 and 10). Our work opens the door to designing systems with bespoke sequence-encoded phase diagrams.

## Materials and methods

### Design of the lattice model and interaction matrix

The interaction matrix includes the following terms: Each interaction domain (SH3 domain or PRM) or explicitly modeled linker bead has a finite v_es_ such that each lattice site may have only one domain or linker bead. All other interactions are nearest neighbor interactions such that adjacent sites *x* and *y* on the lattice are assigned an interaction energy ε*_xy_* in units of *k_B_T*, where *k_B_* is Boltzmann’s constant and *T* is the simulation temperature. We designate lattice sites occupied by SH3 domains using the letter S; sites occupied by PRMs by the letter P; and sites occupying linker beads by the letter L. In the default model, the interaction energies have the form: *u*_SS_ = *u*_PP_ = _uLL_=*u*_SL_ = _uPL_=0 and *u*_SP_ = –2*k_B_T*.

### Design of Monte Carlo moves for simulating the phase behavior of multivalent proteins

Five types of moves were deployed to evolve the system. (i) In addition to occupying adjacent lattice sites, two interacting domains are in a bound state if and only if this is specified by the interaction state of the domains. Accordingly, one of the moves randomly changes the interaction state of a domain without changing lattice positions. (ii) The torsional state of an end module that is tethered on one side is altered and a new interaction state is chosen at random. This attempts to move the module to a new location that is within tethering range of the linker, which is the maximum allowable length for the linker. If the module is an interaction domain, then this move also changes the interaction state of the domain similar to move 1. (iii) Crankshaft motions are applied to modules tethered on both sides. The module is moved to a new location that is within tethering range of all linkers that connect to the module in question. This is followed by randomly choosing a new interaction state if the module is an interaction domain. (iv) This move involves the collective translation of all modules that are part of a connected network. The latter is calculated by analyzing the list of all proteins that are connected through interacting domains. An arbitrary translation in any direction is then attempted. (v) Finally, individual chains are allowed to undergo reptation via a slithering motion of a protein by removing an end domain and its linker and appending it to the other end. The domain and linker are placed in a random position that maintains the tether ranges. After the new position has been assigned, the interaction state of the domain is randomly assigned.

### Acceptance and rejection of Monte Carlo moves

If a move results in placement of a domain or module on a site that is already occupied, then the move is rejected. For rotational, torsional, crankshaft, and reptation moves, the moves that do not lead to steric overlap with occupied sites are accepted according to a modified Metropolis criterion *viz*., min{1,wexp(−ΔE)}. Here, ∆*E* is the change in the energy of the system that results from the proposed move. The energy is normalized with respect to *k_B_T.* The parameter *w* is set based on the proposed type of move. For rotational moves, *w* = 1; for torsional and crankshaft moves, $w=\left(\frac{N_{p}}{N_{c}}\right)$, where *N_p_* and *N_c_* are the number of possible interacting states in the proposed and current states, respectively; finally, for reptation moves, $w=\left(\frac{N_{p}V_{p}}{N_{c}V_{c}}\right)$, where *N_p_* and *N_c_* are the number of possible interacting states in the proposed and current states, respectively whereas *V_p_* and *V_c_* are the total number of conformations the domain and linker could be placed in the proposed state and current state respectively. These modifications to the standard Metropolis Monte Carlo acceptance criterion ensure the preservation of microscopic reversibility. The translation of a connected network does not create or destroy interactions, nor does it move the relevant linkers. Therefore, the proposed translational moves are always accepted if the move does not lead to steric overlaps.

### Production runs to generate phase diagrams

For a majority of the simulations, except those where finite size artifacts were queried or the binding affinities were titrated, the interaction energy between adjacent sites with SH3 domains and PRMs was set to –2*k_B_T*. In every system, there were 2.4 × 10^3^ interaction domains. Concentrations of domains were titrated by changing the number of lattice sites. Each simulation was run for 5 × 10^9^ steps and the average over the last half was used to calculate the size of the largest connected network.

In order to query the onset of a gelation transition, we quantified the fraction of molecules that make up the largest connected cluster within the system. We designate this as ϕ_c_. The value of ϕ_c_ that is associated with crossing the critical concentration for percolation, defined as the gel point, is determined by comparing the largest connected network from a randomly generated network to the critical concentration predicted by Flory-Stockmayer theory. Here, the number of nodes in the random network is set to the number of interaction domains used in the lattice simulations. The random network was generated for stoichiometric concentrations of complementary domains. For each domain of type A, a random number was compared to the gross probability *p* that an individual domain would be interacting with a domain of type B. If the random number was less than *p*, a partner was chosen randomly among the domains of type B that do not already have a binding partner.

### The impact of finite sampling

In order to determine how many Monte Carlo steps the simulations should be run for, we tracked the changes in the largest cluster size for simulations near the critical concentration, where convergence is expected to be the slowest. We then ran our simulations for at least an order of magnitude longer than the equilibration time and analyzed the last half of each simulation to obtain the reported values. For select simulation conditions, we ran independent replicas and reproducibly obtained the same cluster sizes (± < 1%).

### Production runs to generate phase diagrams

In order to locate the concentration where ϕ_c_ exceeds the gel point, we ran simulations using a variety of different sized lattices, ranging from 50 to 340 lattice units. The range of box lengths was incrementally refined until the threshold at which the gel point was crossed could be distinguished at the resolution of a single lattice unit. Under the rare case of statistical ambiguity with respect to this threshold, we ran multiple independent simulations at each box length at the approximate gel point, and then averaged the results over all simulations at each box length to obtain a statistically accurate expected value.

### Calculating the gel points from Flory-Stockmayer theory

The gel point or more precisely, the percolation threshold for multivalent polymers can be estimated by analytical methods, one of which is based on Flory-Stockmayer theories. Here, the important parameters are the number of interacting modules within the polymers, *V*, and the fraction of bound modules, *x*. For a specific multivalent protein that is incorporated into a pre-formed network, the average number of additional proteins recruited into the network is denoted as ε and is expressed as: ε = (*V –* 1)*x*. In a system with two types of multivalent proteins *a* and *b*, such as the poly-SH3 and poly-PRM system, the average number of proteins that are recruited into a pre-formed network of multivalent proteins and their ligands can be expressed as: ε = ε*_a_*ε*_b_* = (*V_a_ –* 1)*x_a_*(*V_b_ –* 1)*x_b_*.

If ε is greater than 1, then on average, each protein that is incorporated into the network will bring more than one additional protein with it thus expanding the network. This cascades into an infinitely large cluster of proteins. However, if ε is less than 1 then the proteins that are added are more likely to terminate the network rather than propagate it. For our synthetic poly-SH3 and poly-PRM system, we can calculate the fraction of interactions through knowledge of the dissociation constant, *K_d_.* We designate the SH3 domains as *a* and the PRMs as *b*. It follows that:

(1) $$K_{d}=\frac{\left(\left[a\right]-\left[ab\right]\right)\left(\left[b\right]-\left[ab\right]\right)}{\left[ab\right]};$$

Here, [*a*], [*b*], and [*ab*] are the concentrations of SH3 domains, PRMs, and bound complexes, respectively. The concentration [*ab*] can be calculated by a simple rearrangement of Equation (1), such that:

(2) $$\left[ab\right]=\frac{\left(\left[a\right]+\left[b\right]+K_{d}-\sqrt{{\left(\left[a\right]+\left[b\right]+d\right)}^{2}-4\left[a\right]\left[b\right]}\right)}{2};$$

Accordingly,

(3) $$\begin{array}{c} x_{a}=\frac{\left[ab\right]}{\left[a\right]}=\frac{\left(\left[a\right]+\left[b\right]+K_{d}-\sqrt{{\left(\left[a\right]+\left[b\right]+d\right)}^{2}-4\left[a\right]\left[b\right]}\right)}{2\left[a\right]},\\ x_{b}=\frac{\left[ab\right]}{\left[b\right]}=\frac{\left(\left[a\right]+\left[b\right]+K_{d}-\sqrt{{\left(\left[a\right]+\left[b\right]+d\right)}^{2}-4\left[a\right]\left[b\right]}\right)}{2\left[b\right]},\\ \text{and }\varepsilon =\frac{\left(\left[a\right]+\left[b\right]+K_{d}-\sqrt{{\left(\left[a\right]+\left[b\right]+d\right)}^{2}-4\left[a\right]\left[b\right]}\right)}{4\left[a\right]\left[b\right]}\left(V_{a}-1\right)\left(V_{b}-1\right); \end{array}$$

We can solve for the percolation threshold or the concentration at the gel point of module *a* as a function of the concentration of module *b* by setting ε = 1. This yields:

(4) $${\left[a\right]}_{c}=\frac{\left[b\right]+\lambda^{2}\left[b\right]-2\lambda K_{d}\pm \left(\lambda +1\right)\sqrt{{\left[b\right]}^{2}{\left(\lambda -1\right)}^{2}-4\lambda K_{d}}}{2\lambda };$$

Here, λ = (*V_a_* – 1)(*V_b_* –1). The percolation threshold can also be calculated for the situation where [*a*] = [*b*]. In this scenario,

(5) $${\left[a\right]}_{c}=\frac{K_{d}\sqrt{\lambda }}{{\left(1-\sqrt{\lambda }\right)}^{2}};$$

**Figure 11.**
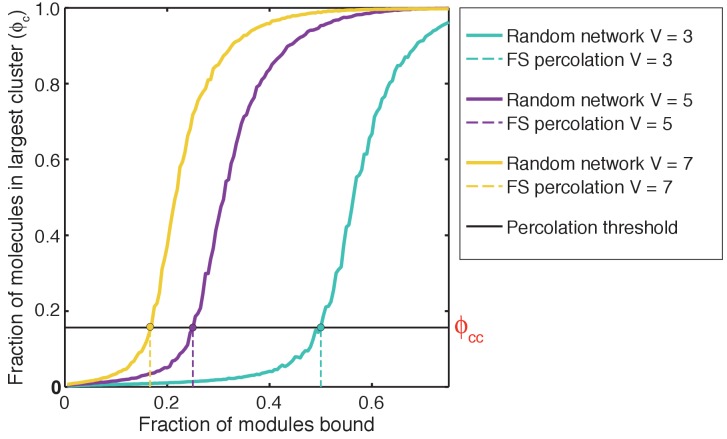
Estimating ϕ_cc_ – the critical value of the fraction of molecules in the largest cluster, ϕ_c_ that defines the gel point: To estimate **ϕ_cc,_** we plot **ϕ_c_** against the fraction of SH3 domains and PRMs that are bound. ϕ_c_ was calculated using a random network model (see Materials and methods) and for a prescribed affinity between interaction domains. ϕ_c_ shows a sigmoidal transition that shifts to the right for systems of lower valence (V). For each system, the dashed vertical lines quantify the percolation thresholds, which refer to the fraction of modules for a given valence *V* that must be bound in order to make a percolated network as prescribed by the theories of Flory and Stockmayer. For a given system of multivalent proteins, the intersection between the solid sigmoidal curve and the dashed vertical line quantifies the value of ϕ_cc_.

We performed simulations of random percolation models that do not account for linkers or the structure of the lattice models. Each simulation takes the valence, the number of multivalent proteins, and the fraction of bound modules as inputs. The value of ϕ_c_ is calculated for prescribed values of the fraction of bound modules and these are shown as solid sigmoidal curves in Figure 11. The theories of Flory (Flory, 1941, 1942b) and Stockmayer (Stockmayer, 1943) can be used to calculate ϕ_cc_ analytically for given values of *V* and the binding energies, as detailed in the Materials and methods section – see Equations (1) – (Zhu and Brangwynne, 2015). These are shown as vertical dashed lines in Figure 11. For a given valence *V*, the horizontal intercept that passes through intersection of the vertical dashed lines and the solid curve defines the value of ϕ_cc_. We find this value to be ≈ 0.17, irrespective of the valence. The concentration of modules at which ϕ_c_ becomes greater than 0.17 is taken to be the value of the gel point *c_g_* for the system of interest. We can calculate the value of *c_g_* directly from our simulations for the multivalent proteins and compare this to the value of *c_g_* that is estimated from Flory-Stockmayer theories.

### Calculation of phase boundaries

We utilized ρ as the order parameter for differentiating between the sol-gel transitions and phase separation. The coexisting concentrations corresponding to the polymer-rich and polymer-poor phases that delineate the two-phase boundary for a given intrinsic affinity between interaction domains were calculated by assuming that the polymer-rich phase is a uniform density sphere and the polymer-poor phase has a uniform density across the remainder of the lattice. The radius of the polymer-rich phase is the radius of the sphere that is the physically relevant root of the equation:

(6) $$\frac{12}{25}\pi N_{T}r_{N}^{5}-\frac{4}{3}N_{T}R_{g}^{2}r_{N}^{3}-\frac{9}{25}N_{N}L^{3}r_{N}^{2}+\frac{\left(N_{N}-N_{T}\right)L^{5}}{4}+N_{T}L^{3}R_{g}^{3}=0;$$

Here, *N_T_* is the total number of proteins in the simulation, *N_N_* is the number of proteins within the largest network, *L* is the lattice length on a side, *R_g_* is the radius of gyration over all the proteins in the simulation, and *r_N_* is the desired radius of the polymer-rich phase. This equation typically admits only one real root that fits within the lattice and this is true for all of our simulations. The phase boundaries were calculated using:

(7) $$c_{sl}=\frac{\left(N_{T}-N_{N}\right)}{\left(L^{3}-\frac{4\pi r_{N}^{3}}{3}\right)N}\;\text{and}\;c_{sh}=\frac{3N_{N}}{4\pi r_{N}^{3}}.$$

### The impact of finite sampling

In addition to starting simulations in the random coil state, we also calculated phase diagrams using simulations that were initialized from a dense phase separated state. For each simulation we equilibrated the proteins in the gel state in a box size of 34 lattice units for 5 × 10^9^ steps. The resulting conformation was then used to initialize simulations in a larger box by expanding the lattice boundary to achieve the desired concentration. For proteins that span the periodic boundary, the first domain was used as the reference for picking which protein image to keep. These initial conditions reproduced the critical concentrations as a function of valence and length.

### All atom simulations

We identified 226 disordered linkers in the human proteome associated with multi-domain proteins. Specifically, we defined disordered linkers in multi-domain proteins as regions predicted to be disordered (Dosztányi et al., 2005) that connected two Pfam domains (Finn et al., 2014) that were predicted or known to be folded. We then filtered for linkers that were between 15 and 200 residues in length, and sub-selected for individual proteins where two or more linkers were found. For each of these sequences all-atom simulations were run to provide a general picture of the global conformational behavior associated with disordered linkers in the human proteome.

In addition to the set of disordered linkers, we also examined fourteen specifically selected sequences, each consisting of 40 residues. These sequences were chosen to enable a titration of conformational properties as a function of the sequence-encoded fraction of charged residues. Sequences of varying charge were extracted randomly from disordered regions in the human proteome. Disordered regions were identified by extracting sequences from the human proteome that were predicted to be disordered by at least five different disorder predictors in the D2P2 database. We required that each stretch have at least 40 consecutive residues that are disordered. We calculated the fraction of residues by tallying the number of ARG, LYS, ASP, and GLU residues in each fragment.

For all sequences described we performed atomistic Monte Carlo simulations using the ABSINTH implicit solvation models and forcefield paradigm (Vitalis and Pappu, 2009b). In this approach, polypeptide chains and solution ions are modeled in atomic detail and the surrounding solvent is modeled using an implicit solvation model that accounts for dielectric inhomogeneities and conformation-specific changes to the free energies of solvation. The simulations were performed and analyzed using tools in the CAMPARI modeling suite (http://campari.sourceforge.net). Forcefield parameters were taken from the abs_opls_3.2.prm parameter set. For each of the fourteen sequences, we performed ten independent simulations, each initialized from a distinct self-avoiding conformation. The methods used to evolve the systems and analyze the simulation results are identical to protocols used in previous studies (Pak et al., 2016 Martin et al., 2016; Das et al., 2016; Das and Pappu, 2013). For simulations of the 226 disordered linkers, five independent simulations per sequence were performed. Each simulation started from a distinct, randomly selected non-overlapping conformation and comprising 5 × 10^6^ equilibration steps and 5 × 10^6^ production steps in 5 mM NaCl. Simulations of the fourteen specifically selected sequences were run for longer to obtain higher resolution statistics.

## Additional files

Supplementary file 1. Excel spreadsheet summarizing the proteome-wide analysis of naturally occurring intrinsically disordered linkers in linear multivalent proteins. Data include the Uniprot ID, the name of the protein from which the linker sequence is drawn, the linker length in terms of number of amino acids, the start and end positions in terms of amino acid numbers for each linker, the disorder score on a scale of 0 to 1, the value of ∆ (see Figure 3), the fraction of positively charged residues (f_+_ or Fpos), the fraction of negatively charged residues (f_–_ or Fneg), location of the diagram-of-states developed by Das and Pappu (Das et al., 2015; Holehouse et al., 2017; Das and Pappu, 2013), amino acid sequence of the linker, Gene Ontology molecular function annotation, Gene Ontology biological process annotation, and Gene Ontology cellular location/component annotation.

Transparent reporting form

## Appendix 1

### Formal definition of v_es_

10.7554/eLife.30294.020 We start with the effective, solvent-mediated potential of mean force, which we denote as *W*(*r*). This is the free energy change associated with bringing a pair of linker residues from a non-interacting reference point to a distance *r* of one another in an aqueous solvent. Therefore, *W*(*r*) quantifies the balance of residue-solvent, solvent-solvent, and residue-residue interactions. If the residues ‘like’ one another more than they ‘like’ the solvent, then the effective inter-residue interactions will be attractive. If the residues ‘like’ the solvent more than they ‘like’ one another, then the effective inter-residue interactions will be repulsive (Rubinstein and Colby, 2003). The probability that a pair of linker residues will be a distance *r* from one another is proportional to the Boltzmann weight exp[–β*W*(*r*)], where β = (*RT*)^–1^, *T* is the temperature and *R* is the ideal gas constant. Because residues cannot sterically overlap with one another, the Boltzmann weight is zero for short inter-residue distances. The Boltzmann weight is one for large separations where the inter-residue interactions are effectively zero. Between these two limits, the Boltzmann weight can be large and positive for separations *r* where the inter-residue interactions are attractive. Conversely, the Boltzmann is negligibly small at inter-residue separations *r* where the effective interactions are repulsive. The effective solvation volume per each pair of residues is defined as the negative of a integral of a function *f*(*r*) (Rubinstein and Colby, 2003; Rubinstein and Semenov, 1998) over the volume available to the pair of residues. Here, *f*(*r*)=exp[–β*W*(*r*)] – 1 and the integral is performed over all pairs of inter-residue separations. Depending on the inter-residue separation *r* and the type of interactions, the *f-*function will be negative (short-range steric overlaps or effective inter-residue repulsions), positive (effective inter-residue attractions), or zero (large separations). The function *f*(*r*) is known as the Mayer *f*-function and the effective solvation volume v_es_ is defined as the negative of the integral of the Mayer *f-*function over the entire volume occupied by the pair of interacting units:

(8) $${\text{v}}_{\text{es}}=-\int d^{3}rf\left(r\right)=\int d^{3}r\left[1-exp\left(\frac{W\left(r\right)}{k_{B}T}\right)\right];$$

The Mayer *f*-function is a dimensionless parameter and the integral in equation (10) has units of volume. It quantifies the two-body or the effective pairwise inter-residue interactions for the polymers in solution. In terms of a virial expansion, at low concentrations, the free energy per unit volume of a polymer solution is written in terms of the polymer concentration as:

(9) $$\frac{F_{\text{solution}}}{V}=\frac{k_{B}T}{2}\left({\text{v}}_{\text{es}}c^{2}+\text{w}c^{3}+\ldots \right);$$

Here, v_es_ has units of volume, and w the three-body interaction coefficient, has units of (volume)^2^ and so on. In dilute concentrations where pairwise interactions dominate, which is the case when v_es_ ≥ 0, it follows that:

(10) $$\frac{F_{\text{soution}}}{V}\approx \frac{k_{B}T{\text{v}}_{\text{es}}c^{2}}{2};$$

The effective interaction energy between residues is negative, zero, or positive depending on the sign of v_es_.
