# Supplementary material for: Intrinsically disordered linkers determine the interplay between phase separation and gelation in multivalent proteins
Source: eLife. 2017 Nov 1;6:e30294. doi: 10.7554/eLife.30294 (PMC5703641; doi:10.7554/eLife.30294)
Supplement: Peer review history

# Decision letter

**DOI:** 10.7554/eLife.30294.021

**Affiliations:** Max Planck Institute of Molecular Cell Biology and GeneticsGermany

In the interests of transparency, eLife includes the editorial decision letter and accompanying author responses. A lightly edited version of the letter sent to the authors after peer review is shown, indicating the most substantive concerns; minor comments are not usually included.

Thank you for submitting your article "Intrinsically disordered linkers determine the interplay between phase separation and gelation in multivalent proteins" for consideration by *eLife*. Your article has been reviewed by two peer reviewers, and the evaluation has been overseen by a Reviewing Editor and Arup Chakraborty as the Senior Editor. The following individual involved in review of your submission has agreed to reveal his identity: Ibrahim I Cisse.

The reviewers have discussed the reviews with one another and the Reviewing Editor has drafted this decision to help you prepare a revised submission.

We feel that your analysis of the factors that drive direct gelation versus phase separation followed by gelation are important for the field. The fact that many physical properties including charge content, changes in solvation volume and length of the linker are key to determining whether continuous gelation or a phase separation + gelation may be expected will be important for further analysis of the molecular basis of phase separation.

There is one issue that was discussed at length, which was the terminology that you use to distinguish phase separation followed by gelation and direct gelation. In my reading of the matter, I think that some of the confusion comes from using the terms continuous and discontinuous. You may want to remove those terms, or find a better way to describe them. To address a broader readership, it could be helpful to add a sketch (e.g. of the free energies) to illustrate the difference between gelation, phase separation and phase separation plus gelation. Please consider the following comments in preparing the revision.

General assessment and major comments:

The authors consider systems composed of multivalent proteins. These proteins can form weak (non-covalent) bonds between each other and thereby potentially form a gel. The authors investigate the physical conditions under which these systems favor to undergo a gel-sol transition, or phase separate first and – due to the higher local density inside the phase separated domain – undergo a gelation transition. They mainly use computer simulations and present phase diagrams, which depict the "sol" phase, the region, where phase separation plus gelation occurs, and where gelation occurs directly. They consider linkers with zero excluded volume (FRC) and linkers with a finite "effective solvation volume" (SARC). One of their main findings (I think) is that the pathway of "phase separation first followed by gelation" is suppressed compared to pathway of "direct gelation" as the "effective solvation volume" is increased.

The Abstract is too general. For instance the statement "...determine the coupling between the phase separation and gelation" sounds too general and in the end means nothing (at least to me). Of course, it is expected that length and sequence-specific features of the linkers will have some effect. Please mention explicitly how they affect the system. What is the qualitative trend when parameter X is changed? And why is this relevant for biology?

Parameters of models: Please give the model and choice of parameters in all captions so that the reader can appreciate which parameters are kept fixed. In addition, please be more explicit and provide a list of significant physical parameters characterizing the system including the linkers. I found it a bit cumbersome to collect all the model details. Maybe a separate section "Model XX" would help. Moreover, please define "effective solvation volume". BTW, is this really the common term (google was not helpful here)? Could you replace it by molecular volume for the purposes of the paper, and if not, please explain in detail? Please explain why the solvation volume can become negative. Please appreciate that the term "effective solvation volume" will not be familiar to most of the readers; a thorough definition is strongly recommended.

Figure 10: What happens to the "phase separation + gelation" regime as the solvation volume is increased? I guess it "just" shifts above 5 kBT, right? If yes, the finding is quantitative; please stress this aspect. Then please make the point why a shift from say 2 kBT to above 5 kBT is indeed significant! If phase separation+ gelation really vanishes, this would be amazing. In this case, please give an explanation.

You provide a clear explanation of the work with interesting phenomena. It is unclear however to what extent their detailed theoretical and computational work will apply to real experimental data. Since the simulations are based on human proteome information, it would be great if the authors can close the loop and discuss in more detail (or at least speculate more on) the implications of their findings for in vivo or in vitro behavior of those proteins. For instance does SH3/PRM (or for example the RGG domain of LAF1) undergo gelation or phase separation + gelation in vitro? In cells? Which proteins have linkers with -0.1<Δ<0.1 and what is their function?

# Reply: Author response

**DOI:** 10.7554/eLife.30294.022

There is one issue that was discussed at length, which was the terminology that you use to distinguish phase separation followed by gelation and direct gelation. In my reading of the matter, I think that some of the confusion comes from using the terms continuous and discontinuous. You may want to remove those terms, or find a better way to describe them. To address a broader readership, it could be helpful to add a sketch (e.g. of the free energies) to illustrate the difference between gelation, phase separation and phase separation plus gelation. Please consider the following comments in preparing the revision.

We agree that the distinction of continuous versus discontinuous transitions is distracting and adds terminological confusion. It is well recognized that phase separation is a first order transition. It would take quite a bit of effort and detract from accessibility to a broad audience to focus on proving that gelation without phase separation is a continuous transition. In fact, this is a technical point that is well beyond the scope of the current contribution. Therefore, we have deleted all mention of the continuity versus discontinuity of the different types of transitions. As for a free energy schematic, Figures 1 and 5 already serve to illustrate the central points we wish to make in this work. Adding another schematic seemed unnecessary and therefore we have not added a figure that may be of limited value to the broader audience of *eLife*.

General assessment and major comments:

The authors consider systems composed of multivalent proteins. These proteins can form weak (non-covalent) bonds between each other and thereby potentially form a gel. The authors investigate the physical conditions under which these systems favor to undergo a gel-sol transition, or phase separate first and – due to the higher local density inside the phase separated domain – undergo a gelation transition. They mainly use computer simulations and present phase diagrams, which depict the "sol" phase, the region, where phase separation plus gelation occurs, and where gelation occurs directly. They consider linkers with zero excluded volume (FRC) and linkers with a finite "effective solvation volume" (SARC). One of their main findings (I think) is that the pathway of "phase separation first followed by gelation" is suppressed compared to pathway of "direct gelation" as the "effective solvation volume" is increased.

The Abstract is too general. For instance the statement "...determine the coupling between the phase separation and gelation" sounds too general and in the end means nothing (at least to me). Of course, it is expected that length and sequence-specific features of the linkers will have some effect. Please mention explicitly how they affect the system. What is the qualitative trend when parameter X is changed? And why is this relevant for biology?

We have rewritten the Abstract and in doing so, we have tried to accommodate the requests made by the reviewer. It is quite possible that the reviewer is asking for a lot to be accomplished within 150 words. However, it is our hope that we have achieved a significant improvement, coming closer to the reviewers’expectations of an Abstract that is not too general, while conveying the relevance for biology.

Parameters of models: Please give the model and choice of parameters in all captions so that the reader can appreciate which parameters are kept fixed.

We thank the reviewers for this suggestion. Table 1 in the revised Materials and methods section includes a table that lists all of the parameters of the model.

In addition, please be more explicit and provide a list of significant physical parameters characterizing the system including the linkers. I found it a bit cumbersome to collect all the model details. Maybe a separate section "Model XX" would help.

In the revised Materials and methods section, we have included a table that the reviewers request.

Moreover, please define "effective solvation volume". BTW, is this really the common term (google was not helpful here)? Could you replace it by molecular volume for the purposes of the paper, and if not, please explain in detail? Please explain why the solvation volume can become negative. Please appreciate that the term "effective solvation volume" will not be familiar to most of the readers; a thorough definition is strongly recommended.

Please note that the original submission had an entire paragraph focused on the defining the effective solvation volume. Additionally, there is an entire appendix that formally describes how the v_es_ becomes negative. We have described everything in gory detail. Please also note that in the polymer physics literature, this term is known as the excluded volume. We recast this as the effective solvation volume because the concept of excluded volume seemed to elude many of our more biologically minded colleagues. This is different from the molecular volume. To respond to the reviewers criticism, we have added new text:

“The effective solvation volumes reflect the average volumes occupied by linkers, referenced to the volume occupied if that linker lacked a bias to be well solvated or poorly solvated (Rubinstein and Colby, 2003). [...] A value of v_es_ ≈ 0 is realized due to a counterbalancing of attractive and repulsive interactions in the linker.”

Figure 10: What happens to the "phase separation + gelation" regime as the solvation volume is increased? I guess it "just" shifts above 5 kBT, right? If yes, the finding is quantitative; please stress this aspect. Then please make the point why a shift from say 2 kBT to above 5 kBT is indeed significant! If phase separation+ gelation really vanishes, this would be amazing. In this case, please give an explanation.

The text states rather explicitly that the gelation without phase separation regime has shifted out of the visible axes.

You provide a clear explanation of the work with interesting phenomena. It is unclear however to what extent their detailed theoretical and computational work will apply to real experimental data. Since the simulations are based on human proteome information, it would be great if the authors can close the loop and discuss in more detail (or at least speculate more on) the implications of their findings for in vivo or in vitro behavior of those proteins. For instance does SH3/PRM (or for example the RGG domain of LAF1) undergo gelation or phase separation + gelation in vitro? In cells? Which proteins have linkers with -0.1<Δ<0.1 and what is their function?

We respectfully submit that we have already engaged in more speculation than we deem appropriate. Supplementary file 1 provides all of the information that the reviewers seek, so we are hard-pressed to know that else is being sought. We believe that our findings are likely to motivate experiments and designs to test our predictions and that these will be performed by the suitable labs who have the requisite expertise. To address the reviewers comments, we have enhanced the Discussion section to offer more direct testable hypotheses. We hope this will suffice.
